# Supplementary material for: Association between Immediate Postoperative Radiographic Findings and Failed Internal Fixation for Trochanteric Fractures: Systematic Review and Meta-Analysis
Source: J Clin Med. 2022 Aug 19;11(16):4879. doi: 10.3390/jcm11164879 (PMC9409751; doi:10.3390/jcm11164879)
Supplement: Supplementary file 1 [file jcm-11-04879-s001.zip › jcm-1830678-supplementary.pdf]

Journal of
Clinical Medicine

MDPI

Online Supplementary Materials

Appendix A. Search strategies

Appendix B Table S1. List of studies excluded from this review and reasons for exclusion

Appendix B Table S2. Characteristics of included studies

Appendix B Table S3. Risk of bias judgment in the included studies

The risk of bias was assessed in 6 domains (study participation, study attrition, prognostic factor measurement, outcome assessment, study confounding, and statistical analysis and reporting) and summarized as overall. The overall risk of bias was rated low if all QUIPS domains were rated low, high if two or more domains were with high risk of bias, and moderate in the other ratings.

Appendix C Figure S1. Forest plots showing the association between poor reduction by Baumgaertner criteria and failed internal fixation

Effect size (ES represented as adjusted odds ratios); CI Confidence interval.ES was determined using the random-effects model weighted by the inverse of the variance estimate. Squares represent ES, with marker size reflecting the statistical weight of the study, obtained using random-effects meta-analysis; horizontal lines represent 95% CIs, diamonds represent the overall odds ratios and 95% CI.

Appendix C Figure S2. Forest plots showing the association between poor reduction by Baumgaertner criteria and cut-out

Effect size (ES represented as adjusted odds ratios); CI Confidence interval.ES was determined using the random-effects model weighted by the inverse of the variance estimate. Squares represent ES, with marker size reflecting the statistical weight of the study, obtained using random-effects meta-analysis; horizontal lines represent 95% CIs, diamonds represent the overall odds ratios and 95% CI.

Appendix C Figure S3. Forest plots showing the association between varus malreduction and failed internal fixation

Effect size (ES represented as adjusted odds ratios); CI Confidence interval.ES was determined using the random-effects model weighted by the inverse of the variance estimate. Squares represent ES, with marker size reflecting the statistical weight of the study, obtained using random-effects meta-analysis; horizontal lines represent 95% CIs, diamonds represent the overall odds ratios and 95% CI.

Appendix C Figure S4. Forest plots showing the association between varus malreduction and cut-out

Effect size (ES represented as adjusted odds ratios); CI Confidence interval.ES was determined using the random-effects model weighted by the inverse of the variance estimate. Squares represent ES, with marker size reflecting the statistical weight of the study, obtained using random-effects meta-analysis; horizontal lines represent 95% CIs, diamonds represent the overall odds ratios and 95% CI.

Appendix C Figure S5. Forest plots showing the association between intramedullary malreduction on anteromedial cortex and failed internal fixation

Effect size (ES represented as adjusted odds ratios); CI Confidence interval.ES was determined using the random-effects model weighted by the inverse of the variance estimate. Squares represent ES, with marker size reflecting the statistical weight of the study, obtained using random-effects meta-analysis; horizontal lines represent 95% CIs, diamonds represent the overall odds ratios and 95% CI.

Appendix C Figure S6. Forest plots showing the association between intramedullary malreduction on anteromedial cortex and cut-out

Effect size (ES represented as adjusted odds ratios); CI Confidence interval.ES was determined using the random-effects model weighted by the inverse of the variance estimate. Squares represent ES, with marker size reflecting the statistical weight of the study, obtained using random-effects meta-analysis; horizontal lines represent 95% CIs, diamonds represent the overall odds ratios and 95% CI.

Appendix C Figure S7. Forest plots showing the association between TAD ≥ 25 and failed internal fixation

Effect size (ES represented as adjusted odds ratios); CI Confidence interval.ES was determined using the random-effects model weighted by the inverse of the variance estimate. Squares represent ES, with marker size reflecting the statistical weight of the study, obtained using random-effects meta-analysis; horizontal lines represent 95% CIs, diamonds represent the overall odds ratios and 95% CI.

Appendix C Figure S8. Forest plots showing the association between TAD ≥ 25 and cut-out

Effect size (ES represented as adjusted odds ratios); CI Confidence interval.ES was determined using the random-effects model weighted by the inverse of the variance estimate. Squares represent ES, with marker size reflecting the statistical weight of the study, obtained using random-effects meta-analysis; horizontal lines represent 95% CIs, diamonds represent the overall odds ratios and 95% CI.

Appendix C Figure S9. Forest plots showing the association between inadequate screw placement in femoral head and failed internal fixation

Effect size (ES represented as adjusted odds ratios); CI Confidence interval.ES was determined using the random-effects model weighted by the inverse of the variance estimate. Squares represent ES, with marker size reflecting the statistical weight of the study, obtained using random-effects meta-analysis; horizontal lines represent 95% CIs, diamonds represent the overall odds ratios and 95% CI.

Appendix C Figure S10. Forest plots showing the association between inadequate screw placement in femoral head and cut-out

Effect size (ES represented as adjusted odds ratios); CI Confidence interval.ES was determined using the random-effects model weighted by the inverse of the variance estimate. Squares represent ES, with marker size reflecting the statistical weight of the study, obtained using random-effects meta-analysis; horizontal lines represent 95% CIs, diamonds represent the overall odds ratios and 95% CI.

Appendix C Figure S11. Funnel plot of estimates from the included studies on the association between poor reduction by Baumgaertner criteria and cut-out

The solid vertical line represents the summary estimate of the association (using random-effects meta-analysis). A significant publication bias was not detected (p = 0.15 for Egger's test). The funnel plot shows asymmetry, that indicates publication bias

Appendix C Figure S12. Funnel plot of estimates from the included studies on the association between TAD ≥ 25 and cut-out

The solid vertical line represents the summary estimate of the association (using random-effects meta-analysis). A significant publication bias was not detected (p = 0.29 for Egger's test). The funnel plot shows symmetry.

Appendix C Figure S13. Funnel plot of estimates from the included studies on the association between inadequate screw placement in femoral head and cut-out

The solid vertical line represents the summary estimate of the association (using random-effects meta-analysis). A significant publication bias was not detected (p = 0.17 for Egger's test). The funnel plot shows asymmetry, that indicates publication bias

Appendix C Figure S14. Subgroup analysis of implant type in the association between poor reduction by Baumgaertner criteria and cut-out

Effect size (ES represented as adjusted odds ratios); CI Confidence interval.ES was determined using the random-effects model weighted by the inverse of the variance estimate. Squares represent ES, with marker size reflecting the statistical weight of the study, obtained using random-effects meta-analysis; horizontal lines represent 95% CIs, diamonds represent the overall odds ratios and 95% CI.

Appendix C Figure S15. Subgroup analysis of implant type in the association between varus malreduction and cut-out

Effect size (ES represented as adjusted odds ratios); CI Confidence interval.ES was determined using the random-effects model weighted by the inverse of the variance estimate. Squares represent ES, with marker size reflecting the statistical weight of the study, obtained using random-effects meta-analysis; horizontal lines represent 95% CIs, diamonds represent the overall odds ratios and 95% CI.

Appendix C Figure S16. Subgroup analysis of implant type in the association between TAD ≥ 25 and failed internal fixation

Effect size (ES represented as adjusted odds ratios); CI Confidence interval.ES was determined using the random-effects model weighted by the inverse of the variance estimate. Squares represent ES, with marker size reflecting the statistical weight of the study,

obtained using random-effects meta-analysis; horizontal lines represent 95% CIs, diamonds represent the overall odds ratios and 95% CI.

Appendix C Figure S17. Subgroup analysis of implant type in the association between TAD ≥ 25 and cut-out

Effect size (ES represented as adjusted odds ratios); CI Confidence interval.ES was determined using the random-effects model weighted by the inverse of the variance estimate. Squares represent ES, with marker size reflecting the statistical weight of the study, obtained using random-effects meta-analysis; horizontal lines represent 95% CIs, diamonds represent the overall odds ratios and 95% CI.

Appendix C Figure S18. Subgroup analysis of implant type in the association between inadequate screw placement in femoral head and failed internal fixation

Effect size (ES represented as adjusted odds ratios); CI Confidence interval.ES was determined using the random-effects model weighted by the inverse of the variance estimate. Squares represent ES, with marker size reflecting the statistical weight of the study, obtained using random-effects meta-analysis; horizontal lines represent 95% CIs, diamonds represent the overall odds ratios and 95% CI.

Appendix C Figure S19. Subgroup analysis of implant type in the association between inadequate screw placement in femoral head and cut-out

Effect size (ES represented as adjusted odds ratios); CI Confidence interval.ES was determined using the random-effects model weighted by the inverse of the variance estimate. Squares represent ES, with marker size reflecting the statistical weight of the study, obtained using random-effects meta-analysis; horizontal lines represent 95% CIs, diamonds represent the overall odds ratios and 95% CI.

Appendix C Table S4. Subgroup analysis on association of immediate postoperative radiographic findings and failed internal fixation; fracture type.

Appendix A. Search strategies

CENTRAL

#1 ("peritrochanteric" NEXT fracture*):ti,ab
#2 ("proximal femoral" NEXT fractures*):ti,ab
#3 ("pertrochanteric" NEXT fracture*):ti,ab
#4 ("trochanteric" NEXT fracture*):ti,ab
#5 ("intertrochanteric femoral" NEXT fracture*):ti,ab
#6 ("intertrochanteric" NEXT fracture*):ti,ab
#7 ("extracapsular hip" NEXT fracture*):ti,ab
#8 mh "hip fractures"
#9 #1 OR #2 OR #3 OR #4 OR #5 OR #6 OR #7 OR #8
#10 "parker s ratio":ti,ab
#11 Cleveland:ti,ab
#12 inferior-center:ti,ab
#13 center-center:ti,ab
#14 (position*:ti,ab OR placement*:ti,ab) AND screw*:ti,ab
#15 ("radiographic" NEXT finding*):ti,ab
#16 "tip apex distance":ti,ab

#17 TAD:ti,ab
#18 "subtype n":ti,ab
#19 "subtype a":ti,ab
#20 "subtype p":ti,ab
#21 ikuta*:ti,ab
#22 "cortical buttress":ti,ab
#23 "cortex support":ti,ab
#24 "cortical support":ti,ab
#25 "medial cortex":ti,ab
#26 "anterior cortex":ti,ab
#27 anteromedial:ti,ab
#28 "collodiaphyseal angle":ti,ab
#29 NSA:ti,ab
#30 "neck shaft angle":ti,ab
#31 ("garden" NEXT alignment*):ti,ab
#32 varus:ti,ab
#33 "tip to apex distance":ti,ab
#34 baumgaertner*:ti,ab
#35 (quality*:ti,ab AND reduction*:ti,ab)
#36 (reduction*:ti,ab AND fracture*:ti,ab))
#37 #10 OR #11 OR #12 OR #13 OR #14 OR #15 OR #16 OR #17 OR #18 OR #19 OR #20 OR #21 OR #22 OR #13 OR #24 OR #25 OR #26 OR #27 OR #28 OR #29 OR #30 OR #31 OR #32 OR #33 OR #34 OR #35 OR #36
#38 #9 AND #37

MEDLINE (via PubMed)

#1 Hip Fractures[mh]
#2 proximal femoral fractures*[tiab]
#3 extracapsular hip fracture*[tiab]
#4 intertrochanteric fracture*[tiab]
#5 intertrochanteric femoral fracture*[tiab]
#6 trochanteric fracture*[tiab]
#7 pertrochanteric fracture*[tiab]
#8 peritrochanteric fracture*[tiab]
#9 #1 OR #2 OR #3 OR #4 OR #5 OR #6 OR #7 OR #8
#10 radiographic finding*[tiab]
#11 quality* [tiab] AND reduction* [tiab]
#12 reduction* [tiab] AND fracture* [tiab]
#13 Baumgaertner*[tiab]
#14 varus[tiab]
#15 Garden alignment*[tiab]
#16 neck shaft angle[tiab]
#17 NSA[tiab]
#18 collodiaphyseal angle[tiab]
#19 anteromedial[tiab]
#20 anterior cortex[tiab]
#21 medial cortex[tiab]
#22 cortical support[tiab]
#23 cortex support[tiab]
#24 cortical buttress[tiab]
#25 Ikuta*[tiab]
#26 subtype P[tiab]
#27 subtype A[tiab]

#28 subtype N[tiab]
#29 TAD[tiab]
#30 tip apex distance[tiab]
#31 tip to apex distance[tiab]
#32 (position*[tiab] or placement*[tiab]) AND screw*[tiab]
#33 center-center [tiab]
#34 inferior-center[tiab]
#35 Cleveland[tiab]
#36 Parker's ratio[tiab]
#37 #10 OR #11 OR #12 OR #13 OR #14 OR #15 OR #16 OR #17 OR #18 OR #19 OR #20 OR #21 OR #22 OR #13 OR #24 OR #25 OR #26 OR #27 OR #28 OR #29 OR #30 OR #31 OR #32 OR #33 OR #34 OR #35 OR #36
#38 #9 AND #37

EMBASE via PROQUEST

S1 (EMB.EXACT.EXPLODE("Hip Fractures"))
S2 (ab(proximal femoral fractures*) OR ti(proximal femoral fractures*))
S3 (ab(extracapsular hip fracture*) OR ti(extracapsular hip fracture*))
S4 (ab(intertrochanteric fracture*) OR ti(intertrochanteric fracture*))
S5 (ab(intertrochanteric femoral fracture*) OR ti(intertrochanteric femoral fracture*))
S6 (ab(trochanteric fracture*) OR ti(trochanteric fracture*))
S7 (ab(pertrochanteric fracture*) OR ti(pertrochanteric fracture*))
S8 (ab(peritrochanteric fracture*) OR ti(peritrochanteric fracture*))
S9 (S1 OR S2 OR S3 OR S4 OR S5 OR S6 OR S7 OR S8)
S10 (ab(radiographic finding*) OR ti(radiographic finding*))
S11 (ab(quality* AND reduction*) OR ti(quality* AND reduction*))
S12 (ab(fracture* AND reduction*) OR ti(fracture* AND reduction*))
S13 (ab(Baumgaertner*) OR ti(Baumgaertner*))
S14 (ab(varus) OR ti(varus))
S15 (ab(Garden alignment*) OR ti(Garden alignment*))
S16 (ab(neck shaft angle) OR ti(neck shaft angle))
S17 (ab(NSA) OR ti(NSA))
S18 (ab(collodiaphyseal angle) OR ti(collodiaphyseal angle))
S19 (ab(anteromedial) OR ti(anteromedial))
S20 (ab(cortical support) OR ti(cortical support))
S21 (ab(anterior cortex) OR ti(anterior cortex))
S22 (ab(medial cortex) OR ti(medial cortex))
S23 (ab(cortex support) OR ti(cortex support))
S24 (ab(cortical buttress) OR ti(cortical buttress))
S25 (ab(Ikuta*) OR ti(Ikuta*))
S26 (ab(subtype N) OR ti(subtype N))
S27 (ab(subtype P) OR ti(subtype P))
S28 (ab(subtype A) OR ti(subtype A))
S29 (ab(TAD) OR ti(TAD))
S30 (ab(tip apex distance) OR ti(tip apex distance))
S31 (EMB.EXACT.EXPLODE("fracture reduction"))
S32 (ab(screw position) OR ti(screw position))
S33 (ab(screw placement) OR ti(screw placement))
S34 (ab(center-center) OR ti(center-center))
S35 (ab(inferior-center) OR ti(inferior-center))
S36 (ab(Cleveland) OR ti(Cleveland))
S37 (ab(Parker's ratio) OR ti(Parker's ratio))

S38 (S10 OR S11 OR S12 OR S13OR S14 OR S15 OR S16 OR S17 OR S18 OR S19 OR S20 OR S21 OR S22 OR S23 OR S24 OR S25 OR S26 OR S27 OR S28 OR S29 OR S30 OR S31 OR S32 OR S33 OR S34 OR S35 OR S36 OR S37 OR S37)

S39 (S9 AND S38)

Clinicaltrials.gov

Condition or disease: intertrochanteric fracture OR trochanteric fracture OR pertrochanteric fracture OR peritrochanteric fracture OR proximal femoral fractures OR intertrochanteric femoral fracture OR intertrochanteric fracture OR extracapsular hip fracture

Other terms: reduction OR parker's ratio OR Cleveland OR radiographic finding OR tip apex distance OR TAD OR medial cortex OR anterior cortex OR anteromedial OR collodiaphyseal angle OR neck shaft angle OR varus

ICTRP

#1 Title ("intertrochanteric fracture" OR "trochanteric fracture" OR "pertrochanteric fracture" OR "extracapsular hip fracture" OR "peritrochanteric fracture")

#2 Conditions: ("radiographic finding" OR "tip apex distance" OR "TAD" OR "neck shaft angle" OR "reduction" OR "varus")

#3 #1 AND #2

Appendix B Table S1. List of studies excluded from this review and reasons for exclusion.

Reason for exclusion	References
Wrong study design	Li S, Chang SM, Niu WX, et al. Comparison of tip apex distance and cut-out complications between helical blades and lag screws in intertrochanteric fractures among the elderly: a meta-analysis. Journal of orthopaedic science. 2015;20(6):1062-1069.

Wrong study design	Tucker A, Warnock M, McDonald S, et al. Fatigue failure of the cephalomedullary nail: revision options, outcomes and review of the literature. European journal of orthopaedic surgery & traumatology. 2018;28(3):511-520.
Wrong study design	Yam M, Kang BJ, Chawla A, et al. Cephalomedullary blade cut-ins: a poorly understood phenomenon. Archives of orthopaedic and trauma surgery. 2020;140(12):1939-1945.
Wrong publication type	Chauveau J, Steinmetz S, Mustaki L, et al. The interest of navigation for the treatment of pertrochanteric fractures with the gamma 3 nail. Swiss Medical Weekly. 2017;34S-35S.
Wrong publication type	Chen SY, Tuladhar R, Chang SM. Fracture Reduction Quality Is More Important Than Implant Choice for Stability Reconstruction in Two-Part Intertrochanteric Femur Fractures. Journal of orthopaedic trauma. 2020;34(6):e227.
Wrong publication type	Garcia M B, Brotat R M, Palencia E J, et al. Cutting out of old age femoral intramedullary nailing. Osteoporosis International. 2013;24(1):S284.
Wrong publication type	George D Al, Afsharpad A, Nwaboku H. Tip apex distance in dynamic hip screw fixation in patients with an extracapsular neck of femur fracture; an audit on change. International Journal of Surgery, 2013;8(11):660.
Wrong publication type	Imerci A, Aydogan NH, Tosun K. The effect on outcomes of the application of circumferential cerclage cable following intramedullary nailing in reverse intertrochanteric femoral fractures. European journal of orthopaedic surgery & traumatology. 2019;29(4):835-842.
Wrong publication type	Kayaokay K, Ozkayin N, Sozbilen M C, et al. Is the tip apex distance a predictor of failure in intertrochanteric hip fractures treated with proximal femoral nail? Injury. 2016;47:S26.
Wrong publication type	Mavrogenis AF, Panagopoulos GN, Megaloikonomos PD, et al. Complications After Hip Nailing for Fractures. Orthopedics. 2016;39(1):e108-e116.
Wrong publication type	Thomson C, Shelton J. The "New Kit Effect": The Impact of Unfamiliar Equipment on Operator Performance in Fractured Neck of Femur Surgery. International Journal of Surgery. 2017;47: S78.
Wrong publication type	Tian KW, Zhang LL, Liu C, et al. The positive, neutral, and negative cortex relationship in fracture reduction of per/inter-trochanteric femur fractures. Journal of orthopaedic trauma. 2020;44(11):2475-2476.

Wrong publication type	Zhou JQ, Chang SM. Failure of PFNA: helical blade perforation and tip-apex distance. Injury. 2012;43(7):1227-1228.
Wrong publication type	Cordero-Ampuero J, Cordero E, Marcos S. et al. Influence of surgical technique quality on mortality, complications and functional result after hip fracture in 915 patients older than 65 years: Poor reduction as a risk factor for mortality. Hip International. 2018;28:154-155.
Wrong participants	Abram SG, Pollard TC, Andrade AJ. Inadequate 'three-point' proximal fixation predicts failure of the Gamma nail. The bone & joint journal. 2013;95-B(6):825-830.
Wrong participants	Akhtar S, Shahab U D, Faiz A S. Frequency of lag screw cutout after dynamic hip screw fixation of stable intertrochanteric femur fracture by keeping tip apex distance less than 25 milli meter. Pakistan Journal of Medical and Health Sciences. 2018;12(2):842-844.
Wrong participants	Albareda-Albareda J, Redondo-Trasobares B, Calvo-Tapies J, et al. Salvage of cephalomedullary nail cutout with the variable angle proximal femoral plate. Injury. 2021;52 Suppl 4:S37-S41.
Wrong participants	Yang H, Zhou F, Tian Y, et al. Analysis of the failure reason of internal fixation in peritrochanteric fractures. Journal of Peking University. Health sciences. 2011;43(5):699-702.
Wrong participants	Appelt A, Suhm N, Baier M, et al. Complications after Intramedullary Stabilization of Proximal Femur Fractures: a Retrospective Analysis of 178 Patients. European journal of trauma and emergency surgery. 2007;33(3):262-267.
Wrong participants	Awan M M Y, Amjad M, Shah A, et al. To study the effectiveness and safety of dynamic hip screw (DHS) fixation for intertrochanteric fractures. Pakistan Journal of Medical and Health Sciences.2019;13(2):342-345.
Wrong participants	Ballal MS, Emms N, Thomas G. Proximal femoral nail failures in extracapsular fractures of the hip. Journal of orthopaedic surgery (Hong Kong). 2008;16(2):146-149.
Wrong participants	Baumgaertner MR, Curtin SL, Lindskog DM, et al. The value of the tip-apex distance in predicting failure of fixation of peritrochanteric fractures of the hip. The Journal of bone and joint surgery. American volume. 1995;77(7):1058-1064.
Wrong participants	Bojan AJ, Beimel C, Taglang G, et al. Critical factors in cut-out complication after Gamma Nail treatment of proximal femoral fractures. BMC musculoskeletal disorders. 2013;14(1).
Wrong participants	Brammar TJ, Kendrew J, Khan RJ, et al. Reverse obliquity and transverse fractures of the trochanteric region of the femur; a review of 101 cases. Injury. 2005;36(7):851-857.
Wrong participants	Chandak R, Malewar N, Jangle A, et al. Description of new "epsilon sign" and its significance in reduction in highly unstable variant of intertrochanteric fracture. European journal of orthopaedic surgery & traumatology. 2019;29(7):1435-1439.

Wrong participants	Chapman T, Zmistowski B, Krieg J, et al. Helical Blade Versus Screw Fixation in the Treatment of Hip Fractures With Cephalomedullary Devices: Incidence of Failure and Atypical "Medial Cutout". Journal of orthopaedic trauma. 2018;32(8):397-402.
Wrong participants	Chen T, Li K, Wang X, et al. Revision cause and effect of gamma nail fixation. Chinese journal of reparative and reconstructive surgery. 2010;24(1):78-81.
Wrong participants	Chikude T, Fujiki E N, De Abreu L C, et al. Cut Out Complications and Anisomelia of the Lower Limbs In Surgery With Valgus Reduction for Intertrochanteric Fractures. International Archives of Medicine, 2015;8(1).
Wrong participants	Choo SK, Oh HK, Ko HT, et al. Effectiveness of controlled telescoping system for lateral hip pain caused by sliding of blade following intramedullary nailing of trochanteric fracture. Injury. 2017;48(10):2201-2206.
Wrong participants	Chu Tong-Bin, Li P. Retrospective analysis of the factors in incidence of postoperative complications in 124 patients with intertrochanteric fracture following the treatment of dynamic hip screw. Chinese Journal of Clinical Rehabilitation. 2004;8(5):832-833.
Wrong participants	Cuervas-Mons M, Mora F, López Fernández J, et al. Desmontaje por efecto cut-out en el enclavado de fracturas pertrocantéreas de fémur: ¿cuál es el tratamiento de rescate de elección? Removal due to cut-out effect in pertrochanteric femoral fractures: what is the rescue treatment of choice? Revista espanola de cirugia ortopedica y traumatologia. 2014;58(6):357-363.
Wrong participants	Cuny C, Scarlat M, Moreau P, et al. Le clou-plaque Staca dans les fractures trochantériennes The Staca nail-plate in the treatment of trochanteric fractures. Revue de chirurgie orthopedique et reparatrice de l'appareil moteur. 1996;82(5):410-416.
Wrong participants	Davis TR, Sher JL, Horsman A, et al. Intertrochanteric femoral fractures. Mechanical failure after internal fixation. The Journal of bone and joint surgery. British volume 1990;72(1):26-31.
Wrong participants	de la Caffinière JY, Ferrer C, Laurent A, et al. Evaluation de l'impaction sur clou-plaque des foyers de fracture pertrochanterienne Evaluation of impaction on bone nail and bone plate of the pertrochanteric fracture. Revue de chirurgie orthopedique et reparatrice de l'appareil moteur. 1997;83(3):243-250.
Wrong participants	Eceviz E, Cevik HB. The V-effect in fixation of intertrochanteric fractures with proximal femoral nails. Orthop Traumatol Surg Res. 2021;107(3).
Wrong participants	Fischer J, Chee Y, Waseem M, et al. IMHS in peritrochanteric fractures. Hip international. 2005;15(1):27-32.
Wrong participants	Flores S A, Woolridge A, Caroom C, et al. The utility of the tip-apex distance in predicting axial migration and cutout with the trochanteric fixation nail system helical blade. Journal of Orthopaedic Trauma, 2016;30(6):e207-e211.
Wrong participants	French BG, Tornetta P 3rd. Use of an interlocked cephalomedullary nail for subtrochanteric fracture stabilization. Clinical orthopaedics and related research. 1998;(348):95-100.
Wrong participants	Gagała J, Kołodziej R, Blacha J, et al. Leczenie złamań blizszej cześci kości udowej przy pomocy ryglowanych pretów śródszpikowych Intramedullary locked nailing in the treatment of proximal femoral fractures. Chirurgia narzadow ruchu i ortopedia polska. 2006;71(3):211-215.

Wrong participants	Geller JA, Saifi C, Morrison TA, et al. Tip-apex distance of intramedullary devices as a predictor of cut-out failure in the treatment of peritrochanteric elderly hip fractures. Journal of orthopaedic trauma. 2010;34(5):719-722.
Wrong participants	Guerra Pinto F, Dantas P, Moreira R, et al. Complications relating to accuracy of reduction of intertrochanteric fractures treated with a compressive hip screw. Hip international. 2010;20(2):221-228.
Wrong participants	Guo JG, Guo JC, Zhao Y, et al. Complications of intertrochanteric fracture treated with dynamic hip screw: cause analysis and prevention. China journal of orthopaedics and traumatology. 2008;21(5):341-342.
Wrong participants	Guo X, Zhang Y, Xiao J, et al. DESIGN AND CLINICAL APPLICATION OF LESSER TROCHANTERIC REDUCTION FIXATION SYSTEM. Chinese journal of reparative and reconstructive surgery. 2015;29(2):133-137.
Wrong participants	Güven M, Yavuz U, Kadioğlu B, et al. Importance of screw position in intertrochanteric femoral fractures treated by dynamic hip screw. Orthop Traumatol Surg Res. 2010;96(1):21-27.
Wrong participants	Guyver P M, McCarthy M J H, Jain N P M, et al. The short-term functional and radiological outcome of patients treated with the Synthes proximal femoral nail antirotation (PFNA) for unstable proximal femoral fractures. European Journal of Orthopaedic Surgery & Traumatology, 2011;21(7):493-501.
Wrong participants	Hampton M, Maripuri N, Khandekar S, et al. Locked rigid antegrade intramedullary nailing of adolescent femoral fractures using a lateral trochanteric entry point. Acta orthopaedica Belgica. 2016;82(4):829-835.
Wrong participants	Han SB, Jung JK, Jang CY, et al. Gamma3 nail with U-Blade (RC) lag screw is effective with better surgical outcomes in trochanteric hip fractures. Scientific reports. 2020;10(1):6021.
Wrong participants	Hao Y, Zhang Z, Zhou F, et al. Risk factors for implant failure in reverse oblique and transverse intertrochanteric fractures treated with proximal femoral nail antirotation (PFNA). Journal of orthopaedic surgery and research. 2019;14(1):350.
Wrong participants	Hernández-Vaquero D, Pérez-Hernández D, Suárez-Vázquez A, et al. Reverse oblique intertrochanteric femoral fractures treated with the gamma nail. Journal of orthopaedic trauma. 2005;29(3):164-167.
Wrong participants	Hoffmann MF, Khoriaty JD, Sietsema DL, et al. Outcome of intramedullary nailing treatment for intertrochanteric femoral fractures. Journal of orthopaedic surgery and research. 2019;14(1):360.
Wrong participants	Honkonen SE, Vihtonen K, Järvinen MJ. Second-generation cephalomedullary nails in the treatment of reverse obliquity intertrochanteric fractures of the proximal femur. Injury. 2004;35(2):179-183.
Wrong participants	Hrubina M, Skoták M, Běhounek J. Komplikace operační léčby zlomenin proximálního femuru metodou DHS Complications of dynamic hip screw treatment for proximal femoral fractures. Acta chirurgiae orthopaedicae et traumatologiae Cechoslovaca. 2010;77(5):395-401.
Wrong participants	İmerci A, Aydogan NH, Tosun K. A comparison of the InterTan nail and proximal femoral fail antirotation in the treatment of reverse intertrochanteric femoral fractures. Acta orthopaedica Belgica. 2018;84(2):123-131.

Wrong participants	Jensen JS, Sonne-Holm S, Tøndevold E. Unstable trochanteric fractures. A comparative analysis of four methods of internal fixation. Acta orthopaedics Scand. 1980;51(6):949-962.
Wrong participants	Jha V, Ahmed T. Modified Short Proximal Femoral Nail for Intertrochanteric Fractures of Femur in Indian Patients - our Experience. Malaysian orthopaedic journal. 2020;14(2):72-82.
Wrong participants	John B, Sharma A, Mahajan A, et al. Tip-apex distance and other predictors of outcome in cephalomedullary nailing of unstable trochanteric fractures. Journal of Clinical Orthopaedics and Trauma. 2019; 10: S88-S94.
Wrong participants	Kang Y F, Xu Y Q, Feng S, et al. Reduction with proximal femur nail anti-rotation in lateral position for unstable femoral intertrochanteric fracture. Chinese Journal of Tissue Engineering Research. 2018;22(19):2988-2993.
Wrong participants	Karapınar L, Turgut A, Kumbaracı M, et al. Evaluation of the quadrants of femoral neck-head in the cephalomedullary fixation of intertrochanteric fractures with a helical blade: Is inferior posterior quadrant also safe? A clinical study. Joint diseases and related surgery. 2021;32(1):93-100.
Wrong participants	Kashigar A, Vincent A, Gunton MJ, et al. Predictors of failure for cephalomedullary nailing of proximal femoral fractures. The bone & joint journal. 2014;96-B(8):1029-1034.
Wrong participants	Kempf I, Grosse A, Taglang G, et al. Gamma nail in the treatment of closed trochanteric fractures. Results and indications of 121 cases. Orthop Traumatol Surg Res. 2014;100(1):75-83.
Wrong participants	Khanna V, Tiwari M. Significance of Tip Apex Distance in Intertrochanteric Fracture femur managed with Proximal femoral nailing. Orthop Traumatol Surg Res. 2021;107(6).
Wrong participants	Kim WY, Han CH, Park JI, et al. Failure of intertrochanteric fracture fixation with a dynamic hip screw in relation to pre-operative fracture stability and osteoporosis. Journal of orthopaedic trauma. 2001;25(6):360-362.
Wrong participants	Kim Y, Bahk WJ, Yoon YC, et al. Radiologic healing of lateral femoral wall fragments after intramedullary nail fixation for A3.3 intertrochanteric fractures. Archives of orthopaedic and trauma surgery. 2015;135(10):1349-1356.
Wrong participants	Korkmaz MF, Erdem MN, Disli Z, et al. Outcomes of trochanteric femoral fractures treated with proximal femoral nail: an analysis of 100 consecutive cases. Clinical interventions in aging. 2014;9:569-574.
Wrong participants	Kovalak E, Ermutlu C, Atay T, et al. Management of unstable pertrochanteric fractures with proximal femoral locking compression plates and affect of neck-shaft angle on functional outcomes. Journal of clinical orthopaedics and trauma. 2017;8(3):209-214.
Wrong participants	Koyuncu Ş, Altay T, Kayalı C, et al. Mechanical failures after fixation with proximal femoral nail and risk factors. Clinical interventions in aging. 2015;10:1959-1965.
Wrong participants	Kozono N, Ikemura S, Yamashita A, et al. Direct reduction may need to be considered to avoid postoperative subtype P in patients with an unstable trochanteric fracture: a retrospective study using a multivariate analysis. Archives of orthopaedic and trauma surgery. 2014;134(12):1649-1654.

Wrong participants	Kraus M, Krischak G, Wiedmann K, et al. Klinische Evaluation des PFNA® und Zusammenhang zwischen Tip-Apex-Distanz und mechanischem Versagen Clinical evaluation of PFNA® and relationship between the tip-apex distance and mechanical failure. Der Unfallchirurg. 2011;114(6):470-478.
Wrong participants	Kuhl M, Beimel C. Does Computer-assisted Surgery Improve Lag Screw Placement During Cephalomedullary Nailing of Intertrochanteric Hip Fractures? Clinical orthopaedics and related research. 2020;478(9):2132-2144.
Wrong participants	Kuhl M, Beimel C. Enhanced cephalomedullary nail lag screw placement and intraoperative tip-apex distance measurement with a novel computer assisted surgery system. Injury. 2016;47(10):2155-2160.
Wrong participants	Kumar GN, Sharma G, Khatri K, et al. Treatment of Unstable Intertrochanteric Fractureswith Proximal Femoral Nail Antirotation II: Our Experience in Indian Patients. The open orthopaedics journal. 2015;9:456-459.
Wrong participants	Kusnezov N, Prabhakar G, Vanden B, et al. Incidence, predictors, and impact of valgus reduction of traumatic intertrochanteric femoral fractures (OTA 31A1-3) treated with the helical blade system: Is anatomic reduction necessary? A retrospective case series. Current Orthopaedic Practice, 2020;31(1):41-47.
Wrong participants	Latif A, Mukherjee K, Ranjan AK, et al. The concept of valgus under reduction in fixation of displaced trochanteric femoral fractures with sliding hip screw. Journal of the Indian Medical Association. 2013;111(12):833-834.
Wrong participants	Lenich A, Mayr E, Rüter A, et al. First results with the trochanter fixation nail (TFN): a report on 120 cases. Archives of orthopaedic and trauma surgery. 2006;126(10):706-712.
Wrong participants	Lenich A, Vester H, Nerlich M, et al. Clinical comparison of the second and third generation of intramedullary devices for trochanteric fractures of the hip--Blade vs screw. Injury. 2010;41(12):1292-1296.
Wrong participants	Leung F, Gudushauri P, Yuen G, et al. Dynamic hip screw blade fixation for intertrochanteric hip fractures. Journal of orthopaedic surgery (Hong Kong). 2012;20(3):302-306.
Wrong participants	Li J, Tang S, Zhang H, et al. Clustering of morphological fracture lines for identifying intertrochanteric fracture classification with Hausdorff distance-based K-means approach. Injury. 2019;50(4):939-949.
Wrong participants	Lorczyński A, Blok K, Kozdryk J, et al. Assessment of functional value of the hip after pertrochanteric fractures treated with dynamic hip screw (DHS). Annales Academiae Medicae Gedanensis. 2016;36:123-131.
Wrong participants	Mainds CC, Newman RJ. Implant failures in patients with proximal fractures of the femur treated with a sliding screw device. Injury. 1989;20(2):98-100.
Wrong participants	Mao W, Ni H, Li L, et al. Comparison of Baumgaertner and Chang reduction quality criteria for the assessment of trochanteric fractures. Bone & joint research. 2019;8(10):502-508.
Wrong participants	Massoud EIE. Fixation of rotationally unstable extracapsular proximal femoral fractures. Ulus Travma Acil Cerrahi Derg. 2018;24(2):168-174.

Wrong participants	Memon K, Siddiqui AM, Khan ZA, et al. Dynamic Hip Screw Fixation Vs. Proximal Femur Nail For Unstable Per-Trochanteric Fractures: A Comparative Analysis Of Outcomes And Complications. J Ayub Med Coll Abbottabad. 2021;33(1):34-38.
Wrong participants	Mereddy P, Kamath S, Ramakrishnan M, et al. The AO/ASIF proximal femoral nail antirotation (PFNA): a new design for the treatment of unstable proximal femoral fractures. Injury. 2009;40(4):428-432.
Wrong participants	Mertl P, Mauger S, Jarde O, et al. Ostéosynthèse des fractures trochantériennes par vis-plaque D.H.S. Etude rétrospective de 223 patients Trochanteric fractures of the femur. A retrospective study of 223 patients treated with the Dynamic Hip Screw. European journal of orthopaedic surgery & traumatology. 1995;5(2):123-127.
Wrong participants	Michael D, Yaniv W, Tal FR, et al. Expandable proximal femoral nail versus gamma proximal femoral nail for the treatment of AO/OTA 31A1-3 fractures. Injury. 2016;47(2):419-423.
Wrong participants	Mir H R, Edwards P, Sanders R, et al. Iatrogenic displacement of minimally or nondisplaced intertrochanteric fractures during intramedullary fixation: incidence, potential etiologies, and clinical impact. Current Orthopaedic Practice. 2013;24(1):58-63.
Wrong participants	Nahm NJ, Frisch NB, Ghacham W, et al. Radiographic Predictors of Screw Cutout for Intertrochanteric Fractures Treated With Cephalomedullary Nails. Journal of surgical orthopaedic advances. 2019;28(2):115-120.
Wrong participants	Nayar SK, Ranjit S, Adebayo O, et al. Implant fracture of the TFNA femoral nail. Journal of clinical orthopaedics and trauma. 2021;22.
Wrong participants	Nikoloski AN, Osbrough AL, Yates PJ. Should the tip-apex distance (TAD) rule be modified for the proximal femoral nail antirotation (PFNA)? A retrospective study. Journal of orthopaedic surgery and research. 2013;8:35.
Wrong participants	Noor F, Nasir H M, Ahmad Shahbaz, et al. Does tip apex distance predict implant failure in stable intertrochanteric fractures of femur? Pakistan Journal of Medical and Health Sciences. 2021;15(3):576-578.
Wrong participants	Nordin S, Zulkifli O, Faisham WI. Mechanical failure of Dynamic Hip Screw (DHS) fixation in intertrochanteric fracture of the femur. The Medical journal of Malaysia. 2001;56 Suppl D:12-17.
Wrong participants	Olséen P, Jonsson B, Ceder L, et al. The Hansson Twin Hook is adequate for fixation of trochanteric fractures: 2 fixation failures in a series of 157 prospectively followed patients. Acta orthopaedica. 2008;79(5):602-608.
Wrong participants	Ozkan K, Eceviz E, Unay K, et al. Treatment of reverse oblique trochanteric femoral fractures with proximal femoral nail. Journal of orthopaedic trauma. 2011;35(4):595-598.
Wrong participants	Pahore M K, Laghari M A, Makhdoom A U, et al. Evaluation of risk of lag screw cut out in unstable intertrochanteric fractures. Journal of the Liaquat University of Medical and Health Sciences, 2011;10:157-162.
Wrong participants	Pajarinen J, Lindahl J, Savolainen V, et al. Femoral shaft medialisation and neck-shaft angle in unstable pertrochanteric femoral fractures. Journal of orthopaedic trauma. 2004;28(6):347-353.

Wrong participants	Palm H, Jacobsen S, Sonne-Holm S, et al. Integrity of the lateral femoral wall in intertrochanteric hip fractures: an important predictor of a reoperation. The Journal of bone and joint surgery. American volume. 2007;89(3):470-475.
Wrong participants	Pang Y, He QF, Zhu LL, et al. Loss of Reduction after Cephalomedullary Nail Fixation of Intertrochanteric Femoral Fracture: A Brief Report. Orthopaedic surgery. 2020;12(6):1998-2003.
Wrong participants	Park CG, Yoon TR, Park KS. Outcomes of Internal Fixation with Compression Hip Screws in Lateral Decubitus Position for Treatment of Femoral Intertrochanteric Fractures. Hip & pelvis. 2018;30(4):254-259.
Wrong participants	Park JH, Lee YS, Park JW, et al. A comparative study of screw and helical proximal femoral nails for the treatment of intertrochanteric fractures. Orthopedics. 2010;33(2):81-85.
Wrong participants	Parry JA, Sapp T, Langford JR, et al. Variables Associated With Lag Screw Sliding After Single-Screw Cephalomedullary Nail Fixation of Intertrochanteric Fractures. Journal of orthopaedic trauma. 2020;34(7):356-358.
Wrong participants	Puthezhath K, Jayaprakash C. Is calcar referenced tip-apex distance a better predicting factor for cutting out in biaxial cephalomedullary nails than tip-apex distance? Journal of orthopaedic surgery (Hong Kong). 2017;25(3).
Wrong participants	Radic R, Yates PJ, Lim TS, et al. 130- versus 135-degree sliding hip screws and failure in pertrochanteric hip fractures. ANZ journal of surgery. 2014;84(12):949-954.
Wrong participants	Rha JD, Kim YH, Yoon SI, et al. Factors affecting sliding of the lag screw in intertrochanteric fractures. Journal of orthopaedic trauma. 1993;17(5):320-324.
Wrong participants	Ruecker AH, Rupprecht M, Gruber M, et al. The treatment of intertrochanteric fractures: results using an intramedullary nail with integrated cephalocervical screws and linear compression. Journal of orthopaedic trauma. 2009;23(1):22-30.
Wrong participants	Sadic S, Custovic S, Jasarevuc M, et al, Krupic F. Proximal Femoral Nail Antirotation in Treatment of Intertrochanteric Hip Fractures: a Retrospective Study in 113 Patients. Medical arch. 2015;69(6):352-356.
Wrong participants	Sahin S, Ertürer E, Oztürk I, et al. Radiographic and functional results of osteosynthesis using the proximal femoral nail antirotation (PFNA) in the treatment of unstable intertrochanteric femoral fractures. Acta orthopaedica et traumatologica turcica. 2010;44(2):127-134.
Wrong participants	Schipper I B, Simmermacher, R K, Hüttl T, et al. Can the proximal femoral nail be improved? European Journal of Trauma. 2005;31(3):258-265.
Wrong participants	Serrano R, Blair JA, Watson DT, et al. Cephalomedullary Nail Fixation of Intertrochanteric Femur Fractures: Are Two Proximal Screws Better Than One?: Erratum. J Orthop Trauma. 2018 Feb;32(2):e75.
Wrong participants	Sevinç HF, Cirpar M, Canbeyli ID, et al. Comparison of functional outcomes in patients fixed with dynamic hip screw and proximal femur nail-anti-rotation in A1 and A2 type intertrochanteric femur fractures. Ulus Travma Acil Cerrahi Derg. 2020;26(5):811-817.

Wrong participants	Seyhan M, Turkmen I, Unay K, et al. Do PFNA devices and Intertan nails both have the same effects in the treatment of trochanteric fractures? A prospective clinical study. Journal of orthopaedic science. 2015;20(6):1053-1061.
Wrong participants	Shabir M, Inam M, Awan AS, et al. Mean Tip-Apex Distance In Lag Screw Cut-Out After Dynamic Hip Screw Fixation Of Hip Fracture. Journal of Ayub Medical College, Abbottabad. 2018;30(3):414-416.
Wrong participants	Shah AA, Kumar S, Rashid RH, et al. Dynamic hip screw fixation for inter-trochanteric fractures: determinants of outcomes. The Journal of the Pakistan Medical Association. 2014;64(12 Suppl 2):S95-S99.
Wrong participants	Sharma A, Mahajan A, John B. A Comparison of the Clinico-Radiological Outcomes with Proximal Femoral Nail (PFN) and Proximal Femoral Nail Antirotation (PFNA) in Fixation of Unstable Intertrochanteric Fractures. Journal of clinical and diagnostic research. 2017;11(7):RC05-RC09.
Wrong participants	Siwach RC, Rohilla R, Singh R, et al. Radiological and functional outcome in unstable, osteoporotic trochanteric fractures stabilized with dynamic helical hip system. Strategies in trauma and limb reconstruction. 2013;8(2):117-122.
Wrong participants	Takai H, Mizuta, K. Murayama M, et al. Comparing the usefulness of a fluoroscopic navigation system in femoral trochanteric fracture for orthopaedic residents with the conventional method. Injury. 2020;51(8):1840-1845.
Wrong participants	Tokgoz M A, Kilicaslan O F. Predictors of Cut-Out after Fixation of Intertrochanteric Fractures with Two Cephalocervical Screwed Proximal Femur Nails. 2021; 32: 287-291.
Wrong participants	Tomás-Hernández J, Núñez-Camarena J, Teixidor-Serra J, et al. Salvage for intramedullary nailing breakage after operative treatment of trochanteric fractures. Injury. 2018;49 Suppl 2:S44-S50.
Wrong participants	Tornetta P, DeHaan A, Hinds D, et al. The Orthopaedic Traumatologist and the Peritrochanteric Hip Fracture-Does Experience Matter? Journal of surgical orthopaedic advances. 2021;30(3):140-143.
Wrong participants	Tucker A, Diamond O, McDonald S, et al. Is there any place for the variable angle proximal femoral plate? A case matched cohort study against the Dynamic Hip Screw system. Injury. 2016;47(10):2173-2181.
Wrong participants	Turgut A, Kalenderer Ö, Günaydın B, et al. Fixation of intertrochanteric femur fractures using Proximal Femoral Nail Antirotation (PFNA) in the lateral decubitus position without a traction table. Acta orthopaedica et traumatologica turcica 2014;48(5):513-520.
Wrong participants	Turgut A, Kalenderer Ö, Karapınar L, et al. Which factor is most important for occurrence of cutout complications in patients treated with proximal femoral nail antirotation? Retrospective analysis of 298 patients. Archives of orthopaedic and trauma surgery. 2016;136(5):623-630.
Wrong participants	Puram C, Pradhan C, Patil A, et al. Outcomes of dynamic hip screw augmented with trochanteric wiring for treatment of unstable type A2 intertrochanteric femur fractures. Injury. 2017;48 Suppl 2:S72-S77.
Wrong participants	Verheyden, A. P., Josten, C. Intramedullary Fixation of Intertrochanteric Fractures with the Proximal Femoral Nail (PFN). Operative Orthopädie und Traumatologie. 2003;15.(1): 20-37.

Wrong participants	Espinosa C, Martinez F, Esteban I et al. Treatment of trochanteric area fractures in elderly patients with Gamma® intramedullary rods. Revista de Ortopedia y Traumatologia. 2000;44(5):434-438.
Wrong participants	Walton MJ, Barnett AJ, Jackson M. Tip-Apex Distance as a Predictor of Failure Following Cephalo-Medullary Fixation for Unstable Fractures of the Proximal Femur. European journal of trauma and emergency surgery. 2008;34(3):273-276.
Wrong participants	Wang H, Wang Y, Yan B, et al. Measuring method of tip-apex distance in treatment of femoral intertrochanteric fracture with proximal femoral nail antirotation. Chinese journal of reparative and reconstructive surgery. 2014;28(1):53-55.
Wrong participants	Wang Q, Yang X, He HZ, et al. Comparative study of InterTAN and Dynamic Hip Screw in treatment of femoral intertrochanteric injury and wound. International journal of clinical and experimental medicine. 2014;7(12):5578-5582.
Wrong participants	Wang YQ, Hu YC, Xu ZM, et al. An intramedullary nail with multifunctional interlocking for all types of fracture in both femurs. Orthopaedic surgery. 2009;1(2):121-126.
Wrong participants	Wang Z, Li K, Gu Z, et al. The risk assessment model of fracture nonunion after intramedullary nailing for subtrochanteric femur fracture. Medicine (Baltimore). 2021;100(12):e25274.
Wrong participants	Warschawski Y, Ankori R, Rutenberg TF, et al. Expandable Proximal Femoral Nail versus Gamma Proximal Femoral Nail for the treatment of hip reverse oblique fractures. Archives of Orthopaedic and Trauma Surgery. 2022;142(5):777-785.
Wrong participants	Watson ST, Schaller TM, Tanner SL, et al. Outcomes of Low-Energy Basicervical Proximal Femoral Fractures Treated with Cephalomedullary Fixation. The Journal of bone and joint surgery. American volume. 2016;98(13):1097-1102.
Wrong participants	Wilson HJ Jr, Rubin BD, Helbig FE, et al. Treatment of interotrochanteric fractures with the Jewett nail: experience with 1,015 cases. Clinical orthopaedics and related research.1980;(148):186-191.
Wrong participants	Windolf J, Hollander DA, Hakimi M, et al. Pitfalls and complications in the use of the proximal femoral nail. Langenbeck's archives of surgery. 2005;390(1):59-65.
Wrong participants	Wolfgang GL, Bryant MH, O'Neill JP. Treatment of intertrochanteric fracture of the femur using sliding screw plate fixation. Clinical orthopaedics and related research. 1982;(163):148-158.
Wrong participants	Wu Y, Watson J T, Kuldjanov D, et al. Rotationally stable fixation for intertrochanteric hip fractures: the Intertan experience, surgical technique, and outcomes. Techniques in Orthopaedics, 2014;29(3):120-132.
Wrong participants	Xiao J, Gao Z, Qin Y, et al. Accuracy of the lesser trochanter for guiding lag screw insertion in hip fracture management. Orthopedics. 2014;37(12):e1080-e1084.
Wrong participants	Xue D, Yu J, Zheng Q, et al. The treatment strategies of intertrochanteric fractures nonunion: An experience of 23 nonunion patients. Injury. 2017;48(3):708-714.

Wrong participants	Yen SH, Lu CC, Ho CJ, et al. Impact of Wedge Effect on Outcomes of Intertrochanteric Fractures Treated with Intramedullary Proximal Femoral Nail. Journal of clinical medicine. 2021;10(21):5112.
Wrong participants	Yoo J, Chang J, Park C, et al. Risk Factors Associated with Failure of Cephalomedullary Nail Fixation in the Treatment of Trochanteric Hip Fractures. Clin Orthopaedic surgery. 2020;12(1):29-36.
Wrong participants	Yoo J, Kim S, Choi J, et al. Gamma 3 U-Blade lag screws in patients with trochanteric femur fractures: are rotation control lag screws better than others? Journal of orthopaedic surgery and research. 2019;14(1):440.
Wrong participants	Zhang W, Antony X R P, Decruz J, et al. Risk factors for mechanical failure of intertrochanteric fractures after fixation with proximal femoral nail antirotation (PFNA II): a study in a Southeast Asian population. Archives of orthopaedic and trauma surgery. 2021; 141(4): 569-575.
Wrong participants	Zhang W, Antony Xavier RP, Decruz J, et al. Risk factors for mechanical failure of intertrochanteric fractures after fixation with proximal femoral nail antirotation (PFNA II): a study in a Southeast Asian population. Archives of orthopaedic and trauma surgery. 2021;141(4):569-575.
Wrong participants	Zhao X, Yan SG, Li H, et al. Short reconstruction nail for intertrochanteric fracture: does it really fit Asian feature? Archives of orthopaedic and trauma surgery. 2012;132(1):81-86.
Wrong outcomes	Aguilar-Alcalá LD, Atri-Levy J, Torres-Gómez A, et al. Factores asociados a fallo en la osteosíntesis de fracturas transtrocantéricas Factor associated in the failure of the ostheosyntesis of transtrocantheric fractures. Acta ortopedica mexicana. 2017;31(4):189-195.
Wrong outcomes	Armagan R, Isik T, Kanar M,et al. Do third generation proximal femur nails have better outcome than second generation nails. Injury. 2013;44:S16.
Wrong outcomes	Goffin JM, Jenkins PJ, Ramaesh R, et al. What is the relevance of the tip-apex distance as a predictor of lag screw cut-out? PLoS One. 2013;8(8).
Wrong outcomes	Holsters L, van Laarhoven S, Bruls RJ, et al. Comparable clinical and radiographical outcomes between second and third generation of gamma nails. Acta orthopaedica Belgica. 2020;86(4):628-635.
Wrong outcomes	Hsu KH, Chang CH, Su YP, et al. Radiographic risk factors for predicting failure of geriatric intertrochanteric fracture treatment with a cephalomedullary nail. Journal of the Chinese Medical Association. 2019;82(7):584-588.
Wrong outcomes	Karataglis D, Boutsiadis A, Papadopoulos P, et al. First experience with a new fixation implant: Technical tips from the clinical application of Proximal Femoral Nail-A II (PFNA-II). Injury. 2011;42:S22.
Wrong outcomes	Lilly RJ, Koueiter DM, Graner KC, et al. Computer-assisted navigation for intramedullary nail fixation of intertrochanteric femur fractures: A randomized, controlled trial. Injury. 2018;49(2):345-350.
Wrong outcomes	Liu W, Wang J, Weaver MJ, et al. Lateral migration with telescoping of a trochanteric fixation nail in the treatment of an intertrochanteric hip fracture. Chinese medical journal. 2014;127(4):680-684.

Wrong outcomes	Shyam Kumar AJ, Parmar V, Bankart J, et al. Comparison of accuracy of lag screw placement in cephalocondylic nails and sliding hip screw plate fixation for extracapsular fractures of the neck of femur. Journal of orthopaedic trauma. 2006;30(5):320-324.
Wrong outcomes	Wang X, Zhang Y, Du S, et al. Reasons of the guide pin eccentricity of helical blade during proximal femoral nail anti-rotation internal fixation for femoral intertrochanteric fractures. Chinese journal of reparative and reconstructive surgery. 2021;35(8):950-955.
Wrong outcomes	Wang Z, Liu Y, Li S, et al. How to get better TAD? Relationship between anteversion angle of nail and position of femoral neck guide pin during nailing of intertrochanteric fractures. BMC musculoskeletal disorders. 2020;21(1).
Wrong outcomes	Yoo JH, Kim TY, Chang JD, et al. Factors influencing functional outcomes in united intertrochanteric hip fractures: a negative effect of lag screw sliding. Orthopedics. 2014;37(12):e1101-e1107.
Wrong intervention	Berkenbaum I, El Banna S. Ostéosynthèse des fractures trochantériennes par plaque de compression percutanée (PC.C.P.) Osteosynthesis of trochanteric fracture with percutaneus compression plate (PC.C.P.). Revue medicale de Bruxelles. 2004;25(1):40-46.
Wrong intervention	Bonicoli E, Andreani L, Piolanti N, et al. Early experience about Anteversa(®) plate for lateral femur fractures. Clinical cases in mineral and bone metabolism. the official journal of the Italian Society of Osteoporosis, Mineral Metabolism, and Skeletal Diseases. 2012;9(3):187-190.
Wrong intervention	Cho M R, Lee J H, Kwon J B, et al. The effect of positive medial cortical support in reduction of pertrochanteric fractures with posteromedial wall defect using a dynamic hip screw. Clinics in Orthopedic Surgery. 2018;10(3):292-298.
Wrong intervention	Galanakis IA, Steriopoulos KA, Dretakis EK. Correct placement of the screw or nail in trochanteric fractures. Effect of the initial placement in the migration. Clinical orthopaedics and related research. 1995;(313):206-213.
Wrong intervention	Jiamton C, Boernert K, Babst R, et al. The nail-shaft-axis of the of proximal femoral nail antirotation (PFNA) is an important prognostic factor in the operative treatment of intertrochanteric fractures. Archives of orthopaedic and trauma surgery. 2018;138(3):339-349.
Wrong intervention	Kazemian GH, Manafi AR, Najafi F, et al. Treatment of intertrochanteric fractures in elderly highrisk patients: dynamic hip screw vs. external fixation. Injury. 2014;45(3):568-572.
Wrong intervention	Knobe M, Drescher W, Heussen N, et al. Is helical blade nailing superior to locked minimally invasive plating in unstable pertrochanteric fractures? Clinical orthopaedics and related research. 2012;470(8):2302-2312.
Wrong intervention	Luo F, Shen J, Xu J, et al. Treatment of AO/OTA 31-A3 intertrochanteric femoral fractures with a percutaneous compression plate. Genetics and molecular research. 2014;69(1):1-7.
Wrong intervention	Othman A. Y. Assessment of a newly designed dynamic condylar screw with an angle of 102° in the treatment of unstable intertrochanteric fractures. European Journal of Orthopaedic Surgery & Traumatology. 2004;14(4):225-229.
Wrong intervention	Schmidt-Rohlfing B, Heussen N, Knobe M, et al. Reoperation rate after internal fixation of intertrochanteric femur fractures with the percutaneous compression plate: what are the risk factors? Journal of orthopaedic trauma. 2013;27(6):312-317.

Wrong intervention	Schmidt-Rohlfing B, Hofman M, Heussen N. Internal fixation of intertrochanteric fractures using the percutaneous compression plate: the event of cutting out and its prediction by the tip-apex-distance. European Orthopaedics and Traumatology. 2015;6(4):393-397.
Wrong intervention	Stover CN, Fish JB, Heap WR. Open reduction of trochanteric fracture. New York state journal of medicine. 1971;71(8):2173-2181.
Wrong intervention	Wang J, Yang T, Kong Q, et al. Treatment of aged intertrochanteric fractures with minimally invasive dynamic hip screws. Chinese journal of reparative and reconstructive surgery. 2009;23(9):1071-1074.
Wrong intervention	Wang JP, Yang TF, Kong QQ, et al. Minimally invasive technique versus conventional technique of dynamic hip screws for intertrochanteric femoral fractures. Archives of orthopaedic and trauma surgery. 2010;130(5):613-620.
Wrong intervention	Yang S, Liu Y, Yang T, et al. Early Clinical Efficacy Comparison Study of Gamma3 Nail, Percutaneous Compression Plate (PCCP) and Femoral Head Replacement (FHR) Treatment on Senile Unstable Intertrochanteric Fractures. Journal of investigative surgery. 2018;31(2):130-135.
Wrong exposures	Albareda J, Laderiga A, Palanca D, et al. Complications and technical problems with the gamma nail. Journal of orthopaedic trauma. 1996;20(1):47-50.
Wrong exposures	Barrios C, Broström LA, Stark A, et al. Healing complications after internal fixation of trochanteric hip fractures: the prognostic value of osteoporosis. Journal of orthopaedic trauma. 1993;7(5):438-442.
Wrong exposures	Carroll L R, Athar M, Shafqat A, et al. Lag screw cut out: Assessing tip-apex distance and a calcar-referenced tip-apex distance. Irish Journal of Medical Science. 2014;183(1):S65.
Wrong exposures	Gadegone WM, Salphale YS. Proximal femoral nail - an analysis of 100 cases of proximal femoral fractures with an average follow up of 1 year. Journal of orthopaedic trauma. 2007;31(3):403-408.
Wrong exposures	Gadegone WM, Salphale YS. Short proximal femoral nail fixation for trochanteric fractures. Journal of orthopaedic surgery (Hong Kong). 2010;18(1):39-44.
Wrong exposures	Gardner MJ, Briggs SM, Kopjar B, et al. Radiographic outcomes of intertrochanteric hip fractures treated with the trochanteric fixation nail. Injury. 2007;38(10):1189-1196.
Wrong exposures	Gundle R, Gargan MF, Simpson AH. How to minimize failures of fixation of unstable intertrochanteric fractures. Injury. 1995;26(9):611-614.
Wrong exposures	Hu S, Du S, Xiong W, et al. Effectiveness of proximal femoral nail anti-rotation for high plane intertrochanteric femur fractures. Chinese journal of reparative and reconstructive surgery. 2021;35(3):307-311.
Wrong exposures	Ibrahim I, Appleton PT, Wixted JJ, et al. Implant cut-out following cephalomedullary nailing of intertrochanteric femur fractures: Are helical blades to blame? Injury. 2019;50(4):926-930.

Wrong exposures	Jain MJ, Mavani KJ, Patel D. Role of Provisional Fixation of Fracture Fragments By Steinmann-Pin and Technical Tips in Proximal Femoral Nailing for Intertrochanteric Fracture. Journal of clinical and diagnostic research. 2017;11(6):RC01-RC05.
Wrong exposures	Kim SS, Kim HJ, Lee CS. Clinical outcomes of PFNA-II in the Asian intertrochanteric fracture patients: Comparison of clinical results according to proximal nail protrusion. Injury. 2020;51(2):361-366.
Wrong exposures	Kumar S, Chadha GN. Dynamic hip screw fixation of intertrochanteric fractures without using traction table. Acta orthopaedica Belgica. 2016;82(2):346-350.
Wrong exposures	Laros GS, Moore JF. Complications of fixation in intertrochanteric fractures. Clinical orthopaedics and related research. 1974;(101):110-119.
Wrong exposures	Larsson S, Friberg S, Hansson LI. Trochanteric fractures. Influence of reduction and implant position on impaction and complications. Clinical orthopaedics and related research. 1990;(259):130-139.
Wrong exposures	Lee YS, Huang HL, Lo TY, et al. Dynamic hip screw in the treatment of intertrochanteric fractures: a comparison of two fixation methods. Journal of orthopaedic trauma. 2007;31(5):683-688.
Wrong exposures	Leung WY, Tsang WL. Conventional muscle-reflection approach versus mini-incision muscle-splitting approach in dynamic hip screw fixation. Journal of orthopaedic surgery (Hong Kong). 2008;16(2):156-161.
Wrong exposures	Li M, Wu L, Liu Y, et al. Clinical evaluation of the Asian proximal femur intramedullary nail antirotation system (PFNA-II) for treatment of intertrochanteric fractures. Journal of orthopaedic surgery and research. 2014;9:112.
Wrong exposures	Liu JJ, Shan LC, Deng BY, et al. Reason and treatment of failure of proximal femoral nail antirotation internal fixation for femoral intertrochanteric fractures of senile patients. Genetics and molecular research. 2014;13(3):5949-5956.
Wrong exposures	Luque Pérez R, Checa Betegón P, Galán-Olleros M, et al. Nailing unstable pertrochanteric fractures: does size matters? Archives of orthopaedic and trauma surgery. 2022;142(1):145-155.
Wrong exposures	Lustenberger A, Bekic J, Ganz R. Rotationsinstabilität trochantärer Femurfrakturen fixiert mit der DHS. Eine radiologische Analyse Rotational instability of trochanteric femoral fractures secured with the dynamic hip screw. A radiologic analysis. Der Unfallchirurg. 1995;98(10):514-517.
Wrong exposures	Madsen JE, Naess L, Aune AK, et al. Dynamic hip screw with trochanteric stabilizing plate in the treatment of unstable proximal femoral fractures: a comparative study with the Gamma nail and compression hip screw. Journal of orthopaedic trauma. 1998;12(4):241-248.
Wrong exposures	Moon N H, Shin W C, Jang J H. Risk factors affecting helical blade cut-through in patients with an intertrochanteric fracture: The anti-sliding effect of the iliotibial band. Journal of Orthopaedic Research. 2017;35.
Wrong exposures	Morihara, T, Arai Y, Tokugawa S, et al. Proximal femoral nail for treatment of trochanteric femoral fractures. Journal of Orthopaedic Surgery. 2007;15(3):273-277.

Wrong exposures	Moroni A, Faldini C, Pegreffi F, et al. Dynamic hip screw compared with external fixation for treatment of osteoporotic pertrochanteric fractures. A prospective, randomized study. The Journal of bone and joint surgery. American volume. 2005;87(4):753-759.
Wrong exposures	Nakata K, Ohzono K, Hiroshima K, et al. Serial change of sliding in intertrochanteric femoral fractures treated with sliding screw system. Archives of orthopaedic and trauma surgery. 1994;113(5):276-280.
Wrong exposures	O'Malley MJ, Kang KK, Azer E, et al. Wedge effect following intramedullary hip screw fixation of intertrochanteric proximal femur fracture. Archives of orthopaedic and trauma surgery. 2015;135(10):1343-1347.
Wrong exposures	Pajarinen J, Lindahl J, Michelsson O, et al. Pertrochanteric femoral fractures treated with a dynamic hip screw or a proximal femoral nail. A randomised study comparing post-operative rehabilitation. The Journal of bone and joint surgery. British volume. 2005;87(1):76-81.
Wrong exposures	Parker MJ. Valgus reduction of trochanteric fractures. Injury. 1993;24(5):313-316.
Wrong exposures	Poyanli OS, Soylemez S, Ozkut AT, et al. Precise placement of lag screws in operative treatment of trochanteric femoral fractures with a new guide system. Injury. 2015;46(11):2190-2195.
Wrong exposures	Qu QG, Wang JH, You XJ. Clinical analysis for fixation failure of 13 patients with intertrochanteric fractures. China journal of orthopaedics and traumatology. 2009;22(9):702-703
Wrong exposures	Schipper IB, Steyerberg EW, Castelein RM, et al. Treatment of unstable trochanteric fractures. Randomised comparison of the gamma nail and the proximal femoral nail. The Journal of bone and joint surgery. British volume 2004;86(1):86-94.
Wrong exposures	Schipper IB, Van Der Werken C. Unstable Trochanteric Fractures and Intramedullary Treatment. European Journal of Trauma. 2004;30(1):29-34.
Wrong exposures	Seo JS, Min HJ, Kim DM, et al. Surgical Results of the Cephalomedullary Nail for the Femoral Intertrochanteric Fracture: Comparison between Non-experienced Surgeons and Experienced Surgeon. Hip & pelvis. 2016;28(4):225-231.
Wrong exposures	Tian K, Liu C, Yan J, et al. Feasibility study of Kirschner wire-fixation-cortical bone technique in treatment of intertrochanteric fracture. Chinese journal of reparative and reconstructive surgery. 2019;33(10):1239-1244.
Wrong exposures	Wagner R, Weckbach A, Sellmair U, et al. Die extraartikuläre proximale Femurfraktur des alten Menschen--DHS oder intramedulläre Hüftschraube zur Frakturversorgung? Extra-articular proximal femur fracture in the elderly--dynamic hip screw or intramedullary hip screw for fracture management? Langenbecks Archiv fur Chirurgie. Supplement. Kongressband. Deutsche Gesellschaft fur Chirurgie. Kongress. 1996;113:963-966.
Wrong exposures	Wang Guo-dong, Yuan Tong-zhou, Xu Nuo, et al. Proximal femoral nail antirotation for intertrochanteric fractures in elderly patients.Chinese Journal of Tissue Engineering Research. 2014;18(17):2679-2684.

Wrong exposures	Wang Z, Hao W, Liu D, et al. Prospective Study of Closed Reduction of Trochanteric Fractures via a Novel Intraoperative Femoral Fracture Reduction Device: Early Clinical Results. Journal of orthopaedic trauma. 2018;32(8):e309-e314.
Wrong exposures	Wei J, Qin DA, Guo XS. Curative effect analysis on proximal frmoral nail antirotation for the treatment of femoral intertrochanteric fracture and integrity of lateral trochanteric wall. China journal of orthopaedics and traumatology. 2015;28(6):572-575.
Wrong exposures	Xu YZ, Geng DC, Mao HQ, et al. A comparison of the proximal femoral nail antirotation device and dynamic hip screw in the treatment of unstable pertrochanteric fracture. The Journal of international medical research. 2010;38(4):1266-1275.
Wrong exposures	Xu Z, Zhang M, Yin J, et al. Redisplacement after reduction with intramedullary nails in surgery of intertrochanteric fracture: cause analysis and preventive measures. Archives of orthopaedic and trauma surgery. 2015;135(6):751-758.
Wrong exposures	Yam M, Chawla C I, Kwek E, et al. Factors in proximal femoral nail antirotation (PFNA) failures. Annals of the Academy of Medicine Singapore. 2015;44(10):S444
Wrong exposures	Yan D, Soon Y, Lv Y, et al. Proximal femoral nail antirotation versus Gamma nail in treatment of femoral trochanteric fractures. Current Orthopaedic Practice. 2012;23(4):346-350.
Wrong exposures	Yu Y, Pan K, Wang G. Femoral trochanteric fracture: PFNA spiral blade placement with the aid of an angler. The Journal of international medical research. 2020;48(3):300060519890782.
Wrong exposures	Zhao F, Guo L, Wang X, et al. Benefit of lag screw placement by a single- or two-screw nailing system in elderly patients with AO/OTA 31-A2 trochanteric fractures. The Journal of international medical research. 2021;49(3).
Wrong exposures	Zheng SN, Yao QQ, Mao FY, et al. Application of 3D printing rapid prototyping-assisted percutaneous fixation in the treatment of intertrochanteric fracture. Experimental and therapeutic medicine. 2017;14(4):3644-3650.
Wrong exposures	Zheng Z, Liu H, Yu X, et al. Clinical study on reduction of difficult-reducing intertrochanteric fracture with ball head screw driver of proximal femoral nail antirotation. Chinese journal of reparative and reconstructive surgery. 2019;33(10):1250-1253.
Wrong exposures	Zhou Bo, Zhu Qi, Zhong Shu-tao, et al. Fixation with three kinds of implants to repair osteoporotic intertrochanteric fractures: univariate and multivariate analysis of failure. Chinese Journal of Tissue Engineering Research. 2015;19(17):2719-2723.
Unclear data	Diamond O, Tucker A, Archbold P. The variable angle martin plate-does a variable angle give variable outcomes over the DHS? A case matched study. HIP International. 2015;25:S115-S116.
Unclear data	Hayward SJ, Lowe LW, Tzevelekos S. Intertrochanteric fractures: a comparison between fixation with a two-piece nail plate and Ender's nails. Journal of orthopaedic trauma. 1983;7(3):153-158.
Unclear data	Limousin A B, Fernandez T E, Arraiz D C, et al. Screw cut-out: Mechanical or biological failure? Osteoporosis International. 2016;27(1):S140.
Unclear data	Parker MJ. Cutting-out of the dynamic hip screw related to its position. The Journal of bone and joint surgery. British volume. 1992;74(4):625.

Unclear data	Singisetti K. Rethinking of tip apex distance with a new cephalomedullary nailing device (PFNA) for hip fracture fixation. HIP International. 2015;25:S65.
Unclear data	Sonmez M M, Camur S, Seckin M F. Treatment strategies for proximal femoral nailing of unstable intertrochanteric fractures; reduction on the traction table or lateral decubitis position without traction table. A prospective randomized trial. Injury. 2013;44:S17.
Unclear data	Thimmaiah R, Ampat G. Continued awareness of tip apex distance in dynamic hip screw. HIP International. 2012;22(4):412.
Unclear data	Uzun M, Ertürer E, Oztürk I,et al. Dengesiz intertrokanterik femur kiriklarinin proksimal femoral çivi ile tedavisi sonrasinda geç dönem radyografik komplikasyonlar ve bunlarin fonksiyonel sonuçlara etkileri Long-term radiographic complications following treatment of unstable intertrochanteric femoral fractures with the proximal femoral nail and effects on functional results. Acta orthopaedica et traumatologica turcicac. 2009;43(6):457-463.
Unclear data	Vidyadhara S, Rao SK. One and two femoral neck screws with intramedullary nails for unstable trochanteric fractures of femur in the elderly--randomised clinical trial. Injury. 2007;38(7):806-814.
Unclear data	Andalib A, Etemadifar M, Yavari P. Clinical Outcomes of Intramedullary and Extramedullary Fixation in Unstable Intertrochanteric Fractures: A Randomized Clinical Trial. The archives of bone and joint surgery. 2020;8(2):190-197.
Unclear data	Apostolopoulos A P, Maris S J, Angelis S, et al. Complications after intramedullary fixation of intertrochanteric fractures. Journal of Musculoskeletal Neuronal Interactions. 2021;21(1):163.
Unclear data	Baik JS, Kim KR, Park BH, et al. Outcomes of Wedge Wing in the Lag Screw for Unstable Intertrochanteric Fractures in Elderly Patients. Hip & pelvis. 2021;33(2):71-77.
Unclear data	Barca P, Pascarella R, Maresca A, et al. Intramedullary nailing's complications of the proximal femur fractures. Journal of Orthopaedics and Traumatology. 2011;12:S92.
Unclear data	Bhatia T, Juyal A, Maheshwari R, et al. Evaluation of Dynamic Hip Screw Blade in Extracapsular Fracture Neck of Femur in the Elderly. Journal of Clinical & Diagnostic Research. 2019;13(6):RC01-RC03.
Unclear data	Bornert K, Beeres F, Jiamton C, et al. Analysis of risk factors for failure of proximal femoral nailing (PFN-A) in intertrochanteric fractures. Swiss Medical Weekly. 2017;147:35S.
Unclear data	Bovbjerg PE, Larsen MS, Madsen CF, et al. Failure of short versus long cephalomedullary nail after intertrochanteric fractures. Journal of orthopaedics. 2019;18:209-212.
Unclear data	Carulli C, Piacentini F, Paoli T, et al. A comparison of two fixation methods for femoral trochanteric fractures: a new generation intramedullary system vs sliding hip screw. Clinical cases in mineral and bone metabolism. 2017;14(1):40-47.
Unclear data	Caruso G, Andreotti M, Pari C, et al. Can TAD and CalTAD predict cut-out after extra-medullary fixation with new generation devices of proximal femoral fractures? A retrospective study. Journal of clinical orthopaedics and trauma. 2017;8(1):68-72.
Unclear data	Catania P, Passaretti D, Montemurro G, et al. Intramedullary nailing for pertrochanteric fractures of proximal femur: a consecutive series of 323 patients treated with two devices. Journal of orthopaedic surgery and research. 2019;14(1):449.
Unclear data	Cetin E, Kılıç M, Kılıç Feyzi, et al. Clinical outcomes of the proximal femoral nails in the treatment of intertrochanteric fractures. Gazi Medical Journal. 2019;30(1):39-42.
Unclear data	Chauveau J, Steinmetz S, Mustaki L, et al. Interest of navigation for the treatment of pertrochanteric fractures with the Gamma 3 nail. Revue de Chirurgie Orthopédique et Traumatologique. 2017;103(7):S109.
Unclear data	Chen P, Fu D. Failure analysis of proximal femoral nail antirotation in treatment of geriatric intertrochanteric fractures. Chinese journal of reparative and reconstructive surgery. 2019;33(10):1270-1274.
Unclear data	Chen Z, Hu C, Zheng Z, et al. Effectiveness of proximal femoral nail anti-rotation combined with minimally invasive percutaneous plate osteosynthesis versus Intertan intramedullary nail fixation in treatment of intertrochanteric fracture with incomplete lateral wall. Chinese journal of reparative and reconstructive surgery. 2020;34(9):1085-1090.

Unclear data Chun YS, Oh H, Cho YJ, et al. Technique and early results of percutaneous reduction of sagittally unstable intertrochateric fractures. Clin Orthopaedic surgery. 2011;3(3):217-224.

Unclear data Ciufo DJ, Ketz JP. Proximal Femoral Shortening and Varus Collapse After Fixation of "Stable" Pertrochanteric Femur Fractures. Journal of orthopaedic trauma. 2021;35(2):87-91.

Unclear data Csonka Á, Ecseri T, Dózsai D, et al. A combfejcsavar helyzetének prognosztikai jelentősége a csípőtáji törések esetén The prognostic value of the hip screw position in intertrochanteric fractures. Orvosi hetilap. 2019;160(9):338-342.

Unclear data Deng HL, Cong YX, Huang H, et al. The Effect of Integrity of Lateral Wall on the Quality of Reduction and Outcomes in Elderly Patients with Intertrochanteric Fracture: A Controlled Study. BioMed research international. 2021.

Unclear data Doğan N, Ertürk C, Gülabi D. Is proximal femoral nailing of unstable intertrochanteric fractures in the lateral decubitus position without a traction table as safe and effective as on a traction table? Injury. 2022;53(2):555-560.

Unclear data Dong Q, Zhang Y G, Tian W. The effect of pfna minimally invasive internal fixation on the postoperative slippage of fixing needle in elderly patients with femoral intertrochanteric fracture and the change of reset image. Acta Medica Mediterranea. 2018;34(3):831-837.

Unclear data Duramaz A, İlter MH. The impact of proximal femoral nail type on clinical and radiological outcomes in the treatment of intertrochanteric femur fractures: a comparative study. European journal of orthopaedic surgery & traumatology. 2019;29(7):1441-1449.

Unclear data Ye PH, Huang L, Zha NF, et al. Proximal femoral nail for the treatment of unstable intertrochanteric femoral fractures. China journal of orthopaedics and traumatology. 2011;24(8):645-647.

Unclear data Fan J, Xu X, Zhou F, et al. Risk factors for implant failure of intertrochanteric fractures with lateral femoral wall fracture after intramedullary nail fixation. Injury. 2021;52(11):3397-3403.

Unclear data Fitzpatrick DC, Sheerin DV, Wolf BR, et al. A randomized, prospective study comparing intertrochanteric hip fracture fixation with the dynamic hip screw and the dynamic helical hip system in a community practice. The Iowa orthopaedic journal. 2011;31:166-172.

Unclear data Fogagnolo F, Kfuri M Jr, Paccola CA. Intramedullary fixation of pertrochanteric hip fractures with the short AO-ASIF proximal femoral nail. Archives of orthopaedic and trauma surgery. 2004;124(1):31-37.

Unclear data Gavaskar AS, Tummala NC, Srinivasan P, et al. Helical Blade or the Integrated Lag Screws: A Matched Pair Analysis of 100 Patients With Unstable Trochanteric Fractures. Journal of orthopaedic trauma. 2018;32(6):274-277.

Unclear data Geraci A, Alberto R, Mingozzi R, et al. The treatment of intertrochanteric fractures of the femur with Endovis nail. Ortop Traumatol Rehabil. 2011;13(6):565-572.

Unclear data Hélin M, Pelissier A, Boyer P, et al. Does the PFNA™ nail limit impaction in unstable intertrochanteric femoral fracture? A 115 case-control series. Orthop Traumatol Surg Res. 2015;101(1):45-49.

Unclear data Ho M, Garau G, Walley G, et al. Minimally invasive dynamic hip screw for fixation of hip fractures. Journal of orthopaedic trauma. 2009;33(2):555-560.

Unclear data Hoffmann R, Schmidmaier G, Schulz R, et al. Classic-Nagel vs. dynamische Hüftschraube (DHS). Eine prospektiv-randomisierte Studie zur Behandlung pertrochantärer Femurfrakturen Classic nail versus DHS. A prospective randomised study of fixation of trochanteric femur fractures. Der Unfallchirurg. 1999;102(3):182-190.

Unclear data Hou Y, Yao Q, Zhang G, et al. Comparative study of proximal femoral shortening after the third generation of Gamma nail versus proximal femoral nail anti-rotation in treatment of intertrochanteric fracture. Chinese journal of reparative and reconstructive surgery. 2018;32(3):338-345.

Unclear data Inglis MRB, Jaarsma RL. Intramedullary hip screw fixation of reverse oblique and transverse trochanteric femur fractures. European Journal of Orthopaedic Surgery & Traumatology. 2008;18(5):323-326.

Unclear data Irvine Jr, James N, Widhalm H. A Comparison of Parker's Ratios for the Treatment of AO/OTA 31-A2 Fractures with Third Generation Gamma Nail or Proximal Femoral Nail Antirotation: Is Central Positioning Always the Best? Journal of the American College of Surgeons. 2014;219(3):S70.

Unclear data	Jagow DM, Yacoubian SV, McCrink EJ 3rd, et al. A novel technique for the fixation of inter-trochantieric hip fractures: A telescoping lag screw. published correction appears in J Orthop. 2020 Dec 15;24:292. Journal of orthopaedics. 2018;15(2):690-694.
Unclear data	Kang JS, Kwon YT, Suh YJ, et al. Outcomes of U-Blade Lag Screw for Cephalomedullary Fixation of Unstable Trochanteric Femur Fractures: A Case Control Study. Geriatr Orthopaedic surgery Rehabil. 2020;11.
Unclear data	Kavroudakis E Karampinas P K, Vlamis J, et al. Does the intramedulary nailing substitute the SHS? 2010;20(3);366.
Unclear data	Kaynak G, Botanlioglu H, Erdal O A, et al. Relation of cephalocervical screw placement and correction loss in intertrochanteric fractures treated with INTERTAN nail. Injury. 2013;44:S15-S16.
Unclear data	Kim Y S, Chung P H, Kang S. et al. Comparative study of compression hip screw and proximal femoral nail in AO/OTA 31-A2.2 intertrochanteric fracture of the femur. HIP International. 2018;28:184-185
Unclear data	Kochai A, Uysal M, Ozalay M, et al. Comparision of PFN and INTERTAN nail for unstable intertrochanteric femoral fracture in mobile patients. International journal of clinical and experimental medicine. 2019;12(5):5468-5474.
Unclear data	Kristan A, Benulič Č, Jaklič M. Reduction of trochanteric fractures in lateral view is significant predictor for radiological and functional result after six months. Injury. 2021;52(10):3036-3041.
Unclear data	Kumar CN, Srivastava MPK. Screw versus helical proximal femoral nail in the treatment of unstable trochanteric fractures in the elderly. Journal of clinical orthopaedics and trauma. 2019;10(4):779-784.
Unclear data	Lambers A, Rieger B, Kop A, et al. Implant Fracture Analysis of the TFNA Proximal Femoral Nail. The Journal of bone and joint surgery. American volume. 2019;101(9):804-811.
Unclear data	Lanzetti RM, Caraffa A, Lupariello D, et al. Comparison between locked and unlocked intramedullary nails in intertrochanteric fractures. European journal of orthopaedic surgery & traumatology. 2018;28(4):649-658.
Unclear data	Laros GS. Intertrochanteric fractures. The role of complications of fixation. Archives of surgery (Chicago, Ill. : 1960). 1975;110(1):37-40.
Unclear data	Lee SR, Kim ST, Yoon MG, et al. The stability score of the intramedullary nailed intertrochanteric fractures: stability of nailed fracture and postoperative patient mobilization. Clin Orthopaedic surgery. 2013;5(1):10-18.
Unclear data	Li M H, Peng H, Liu, Y. Clinical evaluation of asian proximal femoral nail antirotation in treatment of intertrochanteric fractures in the elderly. Journal of the American Geriatrics Society. 2013;61:S330-S331.
Unclear data	Limousin A B, Fernandez T E, Marin P O, Poor biological environment increase the cut out risk in hip fractures. Osteoporosis International. 2019;30:S325.
Unclear data	Limousin B, Fernandez E, Arraiz C, A bad biological enviroment increases the 'cut out' risk in hip fractures. Osteoporosis International. 2018;29(1):S266-S267.
Unclear data	Makki D, Matar HE, Jacob N, et al. Comparison of the reconstruction trochanteric antigrade nail (TAN) with the proximal femoral nail antirotation (PFNA) in the management of reverse oblique intertrochanteric hip fractures. Injury. 2015;46(12):2389-2393.
Unclear data	Mallya S, Kamath SU, Annappa R, et al. The Results of Unstable Intertrochanteric Femur Fracture Treated with Proximal Femoral Nail Antirotation-2 with respect to Different Greater Trochanteric Entry Points. Advances in orthopedics. 2020:2834816.
Unclear data	McCormack R, Panagiotopolous K, Buckley R, et al. A multicentre, prospective, randomised comparison of the sliding hip screw with the Medoff sliding screw and side plate for unstable intertrochanteric hip fractures. Injury. 2013;44(12):1904-1909.
Unclear data	Ngo T H N, Michel-Traverso A, Steinmetz S, et al. Evaluation of the ORTHOFIX CHIMAERA nailing system for trochanteric fracture treatment. In: SWISS MEDICAL WEEKLY. FARNSBURGERSTR 8, CH-4132 MUTTENZ, SWITZERLAND: EMH SWISS MEDICAL PUBLISHERS LTD. 2017;147p:54S-54S.
Unclear data	Okkaoglu M C, Sesen H, Demirkale I. Blade type nail designs cause more varus collaps than screw type nails in the treatment of elderly trochanteric fractures. Injury. 2016;47:S4-S5.

Unclear data	Paganias C, Kontou E, Galanakos S, et al. Report of early results of the use of PFNA II in the treatment of peritrochanteric fractures. Injury. 2011;42:S9.
Unclear data	Pan S, Liu XH, Feng T, et al. Influence of different great trochanteric entry points on the outcome of intertrochanteric fractures: a retrospective cohort study. BMC musculoskeletal disorders. 2017;18(1):107.
Unclear data	Pan S, Lou C. G. Liu C. C., et al. A novel modified trochanteric entry portal and percutaneous technique for Asian patients: a prospective randomized study of the PFNA-II in China. International journal of clinical and experimental medicine. 2017;10(3):4669-4677.
Unclear data	Parry JA, Barrett I, Schoch B, et al. Does the Angle of the Nail Matter for Pertrochanteric Fracture Reduction? Matching Nail Angle and Native Neck-Shaft Angle. Journal of orthopaedic trauma. 2018;32(4):174-177.
Unclear data	Patnaik S, Panda A, Trigen Intertan Nail in Unstable Peritrochanteric femur fractures in the Elderly-functional and radiological outcomes in 50 cases. Orthopaedic Journal of Sports Medicine. 2020;8(5_suppl5).
Unclear data	Ryu HG, Choi YT, Kim SM, et al. A Comparison of U-blade Gamma3 and Gamma3 Nails Used for the Treatment of Intertrochanteric Fractures. Hip & pelvis. 2020;32(1):50-57.
Unclear data	Shi Z, Qiang M, Jia X, et al. Association of the lateral wall integrity with clinical outcomes in older patients with intertrochanteric hip fractures treated with the proximal femoral nail anti-rotation-Asia. Journal of orthopaedic trauma. 2021;45(12):3233-3242.
Unclear data	Song HK, Yoon HK, Yang KH. Presence of a nail in the medullary canal; is it enough to prevent femoral neck shortening in trochanteric fracture? Yonsei medical journal. 2014;55(5):1400-1405.
Unclear data	Sonmez MM, Camur S, Erturer E, et al. Strategies for Proximal Femoral Nailing of Unstable Intertrochanteric Fractures: Lateral Decubitus Position or Traction Table. J Am Acad Orthopaedic surgery. 2017;25(3):e37-e44.
Unclear data	Stern R, Lübbeke A, Suva D, et al. Prospective randomised study comparing screw versus helical blade in the treatment of low-energy trochanteric fractures. International orthopaedics. 2011;35(12):1855-1861.
Unclear data	Su Hao, Kang Sun, Xin Wang. A randomized prospective comparison of Intertan and Gamma3 for treating unstable intertrochanteric fractures. International journal of clinical and experimental medicine. 2016;9(5):8640-8647.
Unclear data	Takigawa N, Moriuchi H, Abe M, et al. Complications and techniques of proximal femoral fractures with the Targon PF. Injury. 2012;43:S18.
Unclear data	Tao R, Lu Y, Xu H, et al. Internal fixation of intertrochanteric hip fractures: a clinical comparison of two implant designs. ScientificWorldJournal. 2013;2013:834825.
Unclear data	Tarrant SM, Graan D, Tarrant DJ, et al. Medial Calcar Comminution and Intramedullary Nail Failure in Unstable Geriatric Trochanteric Hip Fractures. Medicina (Kaunas). 2021;57(4):338.
Unclear data	Turgut A, Günaydin B, Önvural B, et al. Can intertrochanteric femur fractures be nailed properly in lateral decubitus position without traction table: Retrospective analysis of 207 patients. HIP International. 2014;24(5):519.
Unclear data	Valentini R, Martino M, Piovan G, et al. Proximal cut-out in pertrochanteric femural fracture. Acta Biomed. 2014;85(2):144-151.
Unclear data	Valentini R, Zandanel L, Martino M, et al. Proximal cut-out in the pertrochanteric fracture. 2013: s103-s104.
Unclear data	Wang Y, Li Y, Zhang L, et al. Application of three-dimensional visualization technology for digital orthopedics in the reduction and fixation of intertrochanteric fracture. Chinese Journal of Tissue Engineering Research. 2021;25(24):3816-3820.
Unclear data	Whale CS, Hulet DA, Beebe MJ, et al. Cephalomedullary nail versus sliding hip screw for fixation of AO 31 A1/2 intertrochanteric femoral fracture: a 12-year comparison of failure, complications, and mortality. Current orthopaedic practice. 2016;27(6):604-613.
Unclear data	Wong TC, Chiu Y, Tsang WL, et al. A double-blind, prospective, randomised, controlled clinical trial of minimally invasive dynamic hip screw fixation of intertrochanteric fractures. Injury. 2009;40(4):422-427.

Unclear data	Wu CC, Tai CL. Effect of lag-screw positions on modes of fixation failure in elderly patients with unstable intertrochanteric fractures of the femur. Journal of orthopaedic surgery (Hong Kong). 2010;18(2):158-165.
Unclear data	Xiang W, Yejin Z, Yekui Li, et al. Proximal femoral nail antirotation and proximal sliding compression and positive support reduction in the treatment of intertrochanteric fractures in older adults. Chinese Journal of Tissue Engineering Research. 2021;25(27):4361.
Unclear data	Xie Hai-Ming, Jin Zheng-Shuai, Li Yong, et al. Proximal femoral nail antirotation for the treatment of elderly patients with unstable intertrochanteric fractures. Chinese Journal of Tissue Engineering Research. 2012;16(35):6535-6539.
Unclear data	Xu Y, Geng D, Yang H, et al. Treatment of unstable proximal femoral fractures: comparison of the proximal femoral nail antirotation and gamma nail 3. Orthopedics. 2010;33(7):473.
Unclear data	Yang YH, Wang YR, Jiang SD, et al. Proximal femoral nail antirotation and third-generation Gamma nail: which is a better device for the treatment of intertrochanteric fractures? Singapore medical journal. 2013;54(8):446-450.
Unclear data	Yasuda T, Obara S, Hayashi J, et al. Analysis of medial cortical support using postoperative X-ray assessment of reduction and three dimensional multiplanar reconstruction computed tomography images after trochanteric femoral fractures. Orthopaedics and Traumatology: Surgery and Research. 2020;106(4):613-619.
Unclear data	Yu W, Zhang X, Zhu X, et al. A retrospective analysis of the InterTan nail and proximal femoral nail anti-rotation-Asia in the treatment of unstable intertrochanteric femur fractures in the elderly. Journal of orthopaedic surgery and research. 2016;11(1):1-7.
Unclear data	Zehir S, Zehir R, Zehir S, et al. Proximal femoral nail antirotation against dynamic hip screw for unstable trochanteric fractures; a prospective randomized comparison. European journal of trauma and emergency surgery. 2015;41(4):393-400.
Unclear data	Zhang S, Zhang JY, Yang DM, et al. Morphology character and reduction methods of sagittally unstable intertrochanteric fractures. Journal of Peking University. Health sciences. 2017;49(2):236-241.
Unclear data	Zhao C, Liu DY, Guo JJ, et al. Comparison of proximal femoral nail and dynamic hip screw for treating intertrochanteric fractures. China journal of orthopaedics and traumatology. 2009;22(7):535-537.
Unclear data	Aguado-Maestro I, Escudero-Marcos R, García-García JM, et al. Resultados y complicaciones de la osteosíntesis de fracturas pertrocantéreas de fémur mediante clavo endomedular con espiral cefálica (clavo femoral proximal antirrotación) en 200 pacientes Results and complications of pertrochanteric hip fractures using an intramedullary nail with a helical blade (proximal femoral nail antirotation) in 200 patients. Revista espanola de cirugia ortopedica y traumatologia. 2013;57(3):201-207.
Unclear data	Amjad M, Akram R, Zaman A U, et al. Frequency and Causes of Failure of Dynamic Hip Screw Fixation for Interochanteric Fracture. PAKISTAN JOURNAL OF MEDICAL & HEALTH SCIENCES. 2016;10(3):734-740.
Unclear data	Andruszkow H, Frink M, Frömke C, et al. Tip apex distance, hip screw placement, and neck shaft angle as potential risk factors for cut-out failure of hip screws after surgical treatment of intertrochanteric fractures. Journal of orthopaedic trauma. 2012;36(11):2347-2354.
Unclear data	Baek SH, Baek S, Won H, et al. Does proximal femoral nail antirotation achieve better outcome than previous-generation proximal femoral nail? Turkish journal of trauma & emergency surgery. 2020;11(11):483-491.
Unclear data	Boukebous B, Flouzat-Lachaniette CH, Donadio J, et al. Femoral offset loss and internal arch restoration defect are correlated with intramedullary nail cut-out complications after pertrochanteric fractures: a case-control study. European journal of orthopaedic surgery & traumatology. 2019;29(7):1451-1460.
Unclear data	Boukebous B, Guillon P, Vandenbussche E, et al. Correlation between femoral offset loss and dynamic hip screw cut-out complications after pertrochanteric fractures: a case-control study. European journal of orthopaedic surgery & traumatology. 2018;28(7):1321-1326.
Unclear data	Cai Y, Feng J, Chen Y, et al. Comparison of the predictive value of tip-apex distance and calcar referenced tip-apex distance in treatment of femoral intertrochanteric fractures with Asian type proximal femoral nail fixation. Chinese journal of reparative and reconstructive surgery. 2020;34(11):1359-1363.

Unclear data	Chen J, Zuo CH, Zhang CY, et al. Comparison of the effects of two cephalomedullary nails (zimmer natural nail and proximal femoral nail antirotation) in treatment of elderly intertrochan teric fractures. Journal of Peking University. Health sciences. 2019;51(2):283-287.
Unclear data	Dragosloveanu Ș, Dragosloveanu CDM, Cotor DC, et al. long intramedullary nail systems in trochanteric fractures: A randomized prospective single center study. Experimental and therapeutic medicine. 2022;23(1):106.
Unclear data	Fang KB, Lin XC, Shi SJ, et al. Treatment of irreducible femoral intertrochanteric fractures using a wire-guided device. Chinese journal of traumatology. 2021;24(2):104-108.
Unclear data	Herman A, Landau Y, Gutman G, et al. Radiological evaluation of intertrochanteric fracture fixation by the proximal femoral nail. Injury. 2012;43(6):856-863.
Unclear data	Holt G, Nunag P, Duncan K, et al. Outcome after short intramedullary nail fixation of unstable proximal femoral fractures. Acta orthopaedica Belgica. 2010;76(3):347-355.
Unclear data	Hopp S, Wirbel R, Ojodu I, et al. Does the implant make the difference? - Prospective comparison of two different proximal femur nails. Acta orthopaedica Belgica. 2016;82(2):319-331.
Unclear data	Huang JW, Gao XS, Yang YF. Early prediction of implant failures in geriatric intertrochanteric fractures with single-screw cephalomedullary nailing fixation. Injury. 2022;53(2):576-583.
Unclear data	Kumbaraci M, Karapinar L, Turgut A. Comparison of Second and Third-Generation Nails in the Treatment of Intertrochanteric Fracture: Screws versus Helical Blades. The Eurasian journal of medicine. 2017;49(1):7-11.
Unclear data	Lang NW, Breuer R, Beiglboeck H, et al. Migration of the Lag Screw after Intramedullary Treatment of AO/OTA 31.A2.1-3 Pertrochanteric Fractures Does Not Result in Higher Incidence of Cut-Outs, Regardless of Which Implant Was Used: A Comparison of Gamma Nail with and without U-Blade (RC) Lag Screw and Proximal Femur Nail Antirotation (PFNA). J Clin Med. 2019;8(5):615.
Unclear data	Li H, Wang H, Zhang Y, et al. The migration of helical blade and the tip apex distance value in cephalomedullary nail for geriatric intertrochanteric fractures. Chinese journal of reparative and reconstructive surgery. 2019;33(10):1234-1238.
Unclear data	Li J, Cheng L, Jing J. The Asia proximal femoral nail antirotation versus the standard proximal femoral antirotation nail for unstable intertrochanteric fractures in elderly Chinese patients. Orthop Traumatol Surg Res. 2015;101(2):143-146.
Unclear data	Li J, Zhang L, Zhang H, et al. Effect of reduction quality on post-operative outcomes in 31-A2 intertrochanteric fractures following intramedullary fixation: a retrospective study based on computerised tomography findings. Journal of orthopaedic trauma. 2019;43(8):1951-1959.
Unclear data	Liu W, Zhou D, Liu F, et al. Mechanical complications of intertrochanteric hip fractures treated with trochanteric femoral nails. The journal of trauma and acute care surgery. 2013;75(2):304-310.
Unclear data	Lobo-Escolar A, Joven E, Iglesias D, et al. Predictive factors for cutting-out in femoral intramedullary nailing. Injury. 2010;41(12):1312-1316.
Unclear data	Lopes-Coutinho L, Dias-Carvalho A, Esteves N, et al. Traditional distance "tip-apex" vs. new calcar referenced "tip-apex" - which one is the best peritrochanteric osteosynthesis failure predictor? Injury. 2020;51(3):674-677.
Unclear data	Mallya S, Kamath SU, Madegowda A, et al. Comparison of radiological and functional outcome of unstable intertrochanteric femur fractures treated using PFN and PFNA-2 in patients with osteoporosis. European journal of orthopaedic surgery & traumatology. 2019;29(5):1035-1042.
Unclear data	Mao W, He YQ, Tang H, et al. A novel angle on helical blade placement in trochanteric fractures - The axis-blade angle. Injury. 2019;50(7):1333-1338.
Unclear data	Müller F, Doblinger M, Kottmann T, et al. PFNA and DHS for AO/OTA 31-A2 fractures: radiographic measurements, morbidity and mortality. European journal of trauma and emergency surgery. 2020;46(5):947-953.
Unclear data	Palm H, Lysén C, Krasheninnikoff M, et al. Intramedullary nailing appears to be superior in pertrochanteric hip fractures with a detached greater trochanter: 311 consecutive patients followed for 1 year. Acta orthopaedics. 2011;82(2):166-170.

Unclear data	Raghuraman R, Kam JW, Chua DTC. Predictors of failure following fixation of intertrochanteric fractures with proximal femoral nail antirotation. Singapore medical journal. 2019;60(9):463-467.
Unclear data	Schmitz PP, Hannink G, Reijmer J, et al. Increased failure rates after the introduction of the TFNA proximal femoral nail for trochanteric fractures: implant related or learning curve effect? Acta orthopaedics. 2022;93:234-240.
Unclear data	Sever GB, Cankuş MC, Karslı B. Comparison of two different proximal femoral nails in instabil intertrochanteric fractures concerning radiological parameters. İnstabil intertrokanterik kırıklarda iki farklı proksimal femoral çivinin radyolojik parametreler açısından karşılaştırılması. Turkish journal of trauma & emergency surgery. 2021;27(3):344-350.
Unclear data	Shin YS, Chae JE, Kang TW, et al. Prospective randomized study comparing two cephalomedullary nails for elderly intertrochanteric fractures: Zimmer natural nail versus proximal femoral nail antirotation II. Injury. 2017;48(7):1550-1557.
Unclear data	Sun Q, Ge W, Hu H, et al. The Influence of Position of the Displaced Lesser Trochanter on Clinical Outcome of Unstable Trochanteric Femur Fractures in the Elderly. BioMed research international. 2018;2018:5013646.
Unclear data	Tian RH, Zhang QM, Chu FL, et al. Comparison of two methods of locating proximal femoral nail anti-rotation in the treatment of femoral intertrochanteric fractures. Journal of orthopaedic surgery and research. 2020;15(1):108.
Unclear data	Tsai SW, Lin CJ, Tzeng YH, et al. Risk factors for cut-out failure of Gamma3 nails in treating unstable intertrochanteric fractures: An analysis of 176 patients. Journal of the Chinese Medical Association. 2017;80(9):587-594.
Unclear data	Wei W, Gu Z, Cui J, et al. Morphological analysis of coronal femoral intertrochanteric fracture and its effect on reduction and internal fixation. Chinese journal of reparative and reconstructive surgery. 2021;35(9):1093-1099.
Unclear data	Willoughby R. Dynamic hip screw in the management of reverse obliquity intertrochanteric neck of femur fractures. Injury. 2005;36(1):105-109.
Unclear data	Yam M, Chawla A, Kwek E. Rewriting the tip apex distance for the proximal femoral nail anti-rotation. Injury. 2017;48(8):1843-1847.
Unclear data	Yoo J, Kim S, Jung H, et al. Clinical Outcomes of U-blade Gamma3 Nails Used to Treat Patients with Trochanteric Fractures: Retrospective Multicenter Study. Hip & pelvis. 2019;31(2):95-101.
Unclear data	Yoon YC, Oh CW, Sim JA, et al. Intraoperative assessment of reduction quality during nail fixation of intertrochanteric fractures. Injury. 2020;51(2):400-406.
Duplicated data	Fang C, Gudushauri P, Wong TM, et al. Increased Fracture Collapse after Intertrochanteric Fractures Treated by the Dynamic Hip Screw Adversely Affects Walking Ability but Not Survival. BioMed research international. 2016;2016:4175092.
Duplicated data	Stern LC, Gorczyca JT, Kates S, et al. Radiographic Review of Helical Blade Versus Lag Screw Fixation for Cephalomedullary Nailing of Low-Energy Peritrochanteric Femur Fractures: There is a Difference in Cutout. Journal of orthopaedic trauma. 2017;31(6):305-310.
Duplicated data	Yamamoto N, Tamura R, Inoue T, et al. Radiological findings and outcomes of anterior wall fractures in pertrochanteric fractures. Journal of orthopaedic science. 2021;26(2):247-253.
Could not access full text	Bartonícek J, Dousa P, Krbec M. Komplikace osteosyntézy gama-hreben u zlomenin horního konce femuru Complications of osteosynthesis of proximal femur fractures by the gamma nail. Acta chirurgiae orthopaedicae et traumatologiae Cechoslovaca. 1998;65(2):84-89.
Could not access full text	LI Peng, Yang H, Zheng L, et al. Postoperative complications of Dynamic hip screw and its prevention in the treatment of intertrochanteric fracture. Journal of Dalian Medical University. 2009:306-309.

Could not access full text	Pavelka T, Matejka J, Cervenková H. Komplikace osteosyntézy krátkým proximálním femorálním hrebem Complications of internal fixation by a short proximal femoral nail. Acta chirurgiae orthopaedicae et traumatologiae Cechoslovaca. 2005;72(6):344-354.
Background article	Archdeacon MT, Cannada LK, Herscovici D Jr, et al. Prevention of complications after treatment of proximal femoral fractures. Instructional course lectures. 2009;58:13-19.

Appendix B Table S2. Characteristic of included studies.

Study	Methods	Subject characteristics	Exposure used in the present review	Outcomes	Adjusted confounders	Notes
Baumgaertner 1997[1]	Design: retrospective cohort<br>Location: USA<br>Sample size: 316	Age: 78.4<br>Implant: SHS or CMN	TAD	FIF, Cut-out	Unadjusted	Published data
Kawaguchi 1998[2]	Design: retrospective cohort<br>Location: Japan<br>Sample size: 60	Age: 78 (53 to 94)<br>Implant: CMN	Varus malreduction	Cut-out	Unadjusted	Published data
Pervez 2004[3]	Design: retrospective cohort<br>Location: UK<br>Sample size: 100	Age: 81 (median)<br>Implant: SHS	Varus malreduction	Cut-out	Unadjusted	Published data
Hsueh 2010[4]	Design: retrospective cohort<br>Location: People's Republic of China<br>Sample size: 937	Age: N/A<br>Implant: SHS	Baumgaertner reduction criteria, TAD, Screw placement in femoral head	Cut-out	Unadjusted	Published data
De Bruijn 2012[5]	Design: retrospective cohort<br>Location: Netherlands<br>Sample size: 215	Age: 78.0 ± 14.9<br>Implant: SHS or CMN	Baumgaertner reduction criteria	Cut-out	Unadjusted	Published data
Erturer 2012[6]	Design: retrospective cohort<br>Location: Turkey<br>Sample size:36	Age: 70.7 (65 to 96)<br>Implant: CMN	Baumgaertner reduction criteria	FIF, Cut-out	Unadjusted	Published data
Takigawa 2014[7]	Design: retrospective cohort<br>Location: Japan<br>Sample size: 494	Age: 84.3 (51 to 101)<br>Implant: CMN	Reduction on anteromedial cortex	FIF, Cut-out	Unadjusted	Unpublished data
Gunay 2014[8]	Design: retrospective cohort<br>Location: Turkey<br>Sample size: 87	Age: 77 (60 to 96)<br>Implant: CMN	TAD, Screw placement in femoral head	FIF, Cut-out	Unadjusted	Published data and unpublished data
Fang 2015[9]	Design: case control<br>Location: People's Republic	Age: 83.6 (54 to 100)<br>Implant: SHS	Baumgaertner reduction criteria, TAD, Screw	FIF	Age, sex, AO/OTA fracture classification,	Published data

	of China<br>Sample size: 302		placement in femoral head		ASA, days from injury to operation, premorbid walking status, surgeon experience, implants	
Ito 2015[10]	Design: retrospective cohort<br>Location: Japan<br>Sample size: 177	Age: 84 (60 to 97)<br>Implant: CMN	Reduction on anteromedial cortex, TAD	Cut-out	Unadjusted	Unpublished data
Chang 2015[11]	Design: retrospective cohort<br>Location: People's Republic of China<br>Sample size: 127	Age: 81.2 (68 to 97)<br>Implant: CMN	Baumgaertner reduction criteria, Reduction on anteromedial cortex, TAD	FIF, Cut-out	Unadjusted	Published data
Mingo-Robinet 2015[12]	Design: retrospective cohort<br>Location: Spain<br>Sample size: 218	Age: 84.89 ± 7.43<br>Implant: CMN	TAD	Cut-out	Unadjusted	Published data
Temiz 2015[13]	Design: retrospective cohort<br>Location: Turkey<br>Sample size: 32	Age: 72.0 ± 2.9<br>Implant: CMN	Baumgaertner reduction criteria, TAD	FIF, Cut-out	Unadjusted	Published data
Li 2016[14]	Design: prospective cohort<br>Location: People's Republic of China<br>Sample size: 136	Age: 77.2 (50 to 92)<br>Implant: SHS or CMN	Baumgaertner reduction criteria, TAD	Cut-out	Unadjusted	Unpublished data
Karampinas 2016[15]	Design: retrospective cohort<br>Location: Greece<br>Sample size: 126	Age: 80.3 (65 to 92)<br>Implant: CMN	TAD	FIF, Cut-out	Unadjusted	Unpublished data
Hsu 2016[16]	Design: retrospective cohort<br>Location: Taiwan<br>Sample size: 442	Age: N/A<br>Implant: SHS	Varus malreduction, TAD	FIF	AO/OTA fracture classification, lateral view screw position, postoperative lateral wall fracture	Published data
Buyukdogan 2017[17]	Design: retrospective cohort<br>Location: Turkey<br>Sample size: 85	Age: 77.0 ± 9.6<br>Implant: CMN	Baumgaertner reduction criteria	Cut-out	TAD, Baumgaertner reduction criteria	Published data
Fujii 2017[18]	Design: case control<br>Location: Japan<br>Sample size: 59	Age: 84.7 (77 to 90)<br>Implant: CMN	Reduction on anteromedial cortex, TAD, Screw placement in femoral head	Cut-out	Age, sex, AO/OTA fracture classification	Published data and unpublished data
Caruso 2017[19]	Design: retrospective cohort	Age: 84.1 ± 6.3<br>Implant: CMN	TAD, Screw placement in femoral head	Cut-out	Age, sex, AO/OTA fracture classification	Unpublished data

	Location: Italy<br>Sample size: 571					
Ciufo 2017[20]	Design: retrospective cohort<br>Location: USA<br>Sample size: 362	Age: 83 ± (55 to 102)<br>Implant: CMN	Varus malreduction, TAD	Cut-out	Lateral wall fracture, posteromedial fragment, basicervical gap, screw superior to mid-neck	Published data
Morvan 2018[21]	Design: retrospective cohort<br>Location: France<br>Sample size: 228	Age: 87<br>Implant: SHS or CMN	Baumgaertner reduction criteria	Cut-out	Sex, Parker's Ratio Method	Published data
Aicale 2018[22]	Design: retrospective cohort<br>Location: Italy<br>Sample size: 68	Age: 86 ± 19<br>Implant: CMN	TAD, screw placement in femoral head	Cut-out	Unadjusted	Published data
Murena 2018[23]	Design: retrospective cohort<br>Location: Italy<br>Sample size: 813	Age: 84.7 ± 7.0<br>Implant: CMN	Baumgaertner reduction criteria	Cut-out	Parker AP	Published data
Kim 2018[24]	Design: retrospective cohort<br>Location: Korea<br>Sample size: 345	Age: 82.6 (60 to 109)<br>Implant: CMN	Baumgaertner reduction criteria	Cut-out	Sex, implant	Published data
Swaroop 2020[25]	Design: prospective cohort<br>Location: India<br>Sample size: 61	Age: 75.39 ± 8.4<br>Implant: CMN	Baumgaertner reduction criteria, Screw placement in femoral head	Cut-out	Unadjusted	Published data
Hancioglu 2020[26]	Design: retrospective cohort<br>Location: Turkey<br>Sample size: 65	Age: 75.9 (65 to 95)<br>Implant: CMN	Screw placement in femoral head	Cut-out	Unadjusted	Published data
Jamshad 2021[27]	Design: prospective cohort<br>Location: India<br>Sample size: 35	Age: 65.6 (60 to 84)<br>Implant: CMN	Varus malreduction, TAD	Cut-out	Unadjusted	Published data
Barlow 2021[28]	Design: retrospective cohort<br>Location: Poland<br>Sample size: 53	Age: N/A<br>Implant: SHS	Screw placement in femoral head	FIF, Cut-out	Unadjusted	Published data
Cordero-Ampuero 2021[29]	Design: retrospective cohort<br>Location: Spain<br>Sample size: 795	Age: 85.66 ± 7.13<br>Implant: SHS or CMN	TAD	Cut-out	Unadjusted	Published data
Çepni 2021[30]	Design: retrospective cohort<br>Location: Turkey<br>Sample size: 125	Age: 78.3 (65 to 95)<br>Implant: CMN	Baumgaertner reduction criteria, Screw placement in femoral head	FIF, Cut-out	Age, sex, tip–neck distance ratio	Published data
Yamamoto 2021[31]	Design: retrospective cohort	Age: 82.9 ± 8.3<br>Implant: CMN	Baumgaertner reduction criteria, Varus malreduction,	FIF, Cut-out	Age, sex, AO/OTA fracture classification	Published data and

	Location: Japan<br>Sample size: 390		TAD, Screw placement in femoral head			unpublished data
Momii 2021[32]	Design: retrospective cohort<br>Location: Japan<br>Sample size: 59	Age: 82.9 (60 to 99)<br>Implant: SHS or CMN	Reduction on anteromedial cortex, TAD	FIF, Cut-out	Unadjusted	Unpublished data
Hwang 2021[33]	Design: retrospective cohort<br>Location: Korea<br>Sample size: 366	Age: 75 (63 to 83)<br>Implant: SHS or CMN	Baumgaertner reduction criteria, Varus malreduction	Cut-out	Unadjusted	Published data
Shon 2021[34]	Design: retrospective cohort<br>Location: Korea<br>Sample size: 94	Age: 77.6 ± 7.8<br>Implant: CMN	Reduction on anteromedial cortex, TAD, Screw placement in femoral head	FIF, Cut-out	Age, sex, AO/OTA fracture classification	Published data and unpublished data
Yamamoto 2022[35]	Design: retrospective cohort<br>Location: Japan<br>Sample size: 299	Age: 83.1 ± 8.2<br>Implant: CMN	Baumgaertner reduction criteria, Reduction on anteromedial cortex	FIF,<br>Cut-out	Unadjusted	Published data
Goto 2022[36]	Design: retrospective cohort<br>Location: Japan<br>Sample size: 263	Age: 84.0 ± 7.4<br>Implant: CMN	Reduction on anteromedial cortex	FIF, Cut-out	Unadjusted	Unpublished data

Numbers are displayed as mean ± SD or mean (range). SHS, sliding hip screw; CMN, cephalomedullary nail; TAD, tip-apex distance; FIF, failed internal fixation; AO/OTA, Arbeitsgemeinschaft für Osteosynthesefragen/Orthopedic Trauma Association; AP, anteroposterior; ASA, American Society of *Anesthesiology* Physical Status Classification System; N/A, not applicable

1. Baumgaertner MR, Solberg BD. Awareness of tip-apex distance reduces failure of fixation of trochanteric fractures of the hip. J Bone Joint Surg Br. 1997;79(6):969-971.
2. Kawaguchi S, Sawada K, Nabeta Y. Cutting-out of the lag screw after internal fixation with the Asiatic gamma nail. Injury. 1998;29(1):47-53.
3. Pervez H, Parker MJ, Vowler S. Prediction of fixation failure after sliding hip screw fixation. Injury. 2004;35(10):994-998.
4. Hsueh KK, Fang CK, Chen CM, Su YP, Wu HF, Chiu FY. Risk factors in cutout of sliding hip screw in intertrochanteric fractures: an evaluation of 937 patients. Int Orthop. 2010;34(8):1273-1276.
5. De Bruijn K, den Hartog D, Tuinebreijer W, Roukema G. Reliability of predictors for screw cutout in intertrochanteric hip fractures. J Bone Joint Surg Am. 2012;94(14):1266-1272.
6. Erturer RE, Sonmez MM, Sari S, Seckin MF, Kara A, Ozturk I. Intramedullary osteosynthesis of instable intertrochanteric femur fractures with Profin(R) nail in elderly patients. Acta Orthop Traumatol Turc. 2012;46(2):107-112.
7. Takigawa N, Moriuchi H, Abe M, Yasui K, Eshiro H, Kinoshita M. Complications and fixation techniques of trochanteric fractures with the TARGON((R)) PF. Injury. 2014;45 Suppl 1:S44-48.
8. Gunay C, Atalar H, Altay M, Yavuz O, Yildirim A, Oken O. Does the wedge wing in the neck screw prevent cut-out failure in OTA/AO 31- A2 trochanteric fractures in elderly patients? Acta Orthop Belg. 2014;80(1):26-33.

9. Fang C, Lau TW, Wong TM, Lee HL, Leung F. Sliding hip screw versus sliding helical blade for intertrochanteric fractures. Bone Joint J. 2015;97-B(3):398-404.
10. Ito J, Takakubo Y, Sasaki K, Sasaki J, Owashi K, Takagi M. Prevention of excessive postoperative sliding of the short femoral nail in femoral trochanteric fractures. Arch Orthop Trauma Surg. 2015;135(5):651-657.
11. Chang SM, Zhang YQ, Ma Z, Li Q, Dargel J, Eysel P. Fracture reduction with positive medial cortical support: a key element in stability reconstruction for the unstable pertrochanteric hip fractures. Arch Orthop Trauma Surg. 2015;135(6):811-818.
12. Mingo-Robinet J, Torres-Torres M, Martinez-Cervell C, et al. Comparative study of the second and third generation of gamma nail for trochanteric fractures: review of 218 cases. J Orthop Trauma. 2015;29(3):e85-90.
13. Temiz A, Durak A, Atici T. Unstable intertrochanteric femur fractures in geriatric patients treated with the DLT trochanteric nail. Injury. 2015;46 Suppl 2:S41-46.
14. Li C, Xie B, Chen S, Lin G, Yang G, Zhang L. The effect of local bone density on mechanical failure after internal fixation of pertrochanteric fractures. Arch Orthop Trauma Surg. 2016;136(2):223-232.
15. Karampinas P, Vlamis J, Papadelis E, Pneumaticos S. Clinical Outcome of Peritrochanteric Fractures Treatment with an Antegrade Trochanteric Static Lag Screw Nail (Inter TAN). British Journal of Medicine and Medical Research. 2016;18(11):1-7.
16. Hsu CE, Huang KC, Lin TC, Tong KM, Lee MH, Chiu YC. Integrated risk scoring model for predicting dynamic hip screw treatment outcome of intertrochanteric fracture. Injury. 2016;47(11):2501-2506.
17. Buyukdogan K, Caglar O, Isik S, Tokgozoglu M, Atilla B. Risk factors for cut-out of double lag screw fixation in proximal femoral fractures. Injury. 2017;48(2):414-418.
18. Fujii T, Nakayama S, Hara M, Koizumi W, Itabashi T, Saito M. Tip-Apex Distance Is Most Important of Six Predictors of Screw Cutout After Internal Fixation of Intertrochanteric Fractures in Women. JB JS Open Access. 2017;2(4):e0022.
19. Caruso G, Bonomo M, Valpiani G, et al. A six-year retrospective analysis of cut-out risk predictors in cephalomedullary nailing for pertrochanteric fractures: Can the tip-apex distance (TAD) still be considered the best parameter? Bone Joint Res. 2017;6(8):481-488.
20. Ciufo DJ, Zaruta DA, Lipof JS, Judd KT, Gorczyca JT, Ketz JP. Risk Factors Associated With Cephalomedullary Nail Cutout in the Treatment of Trochanteric Hip Fractures. J Orthop Trauma. 2017;31(11):583-588.
21. Morvan A, Boddaert J, Cohen-Bittan J, Picard H, Pascal-Mousselard H, Khiami F. Risk factors for cut-out after internal fixation of trochanteric fractures in elderly subjects. Orthop Traumatol Surg Res. 2018;104(8):1183-1187.
22. Aicale R, Maffulli N. Greater rate of cephalic screw mobilisation following proximal femoral nailing in hip fractures with a tip-apex distance (TAD) and a calcar referenced TAD greater than 25 mm. J Orthop Surg Res. 2018;13(1):106.
23. Murena L, Moretti A, Meo F, et al. Predictors of cut-out after cephalomedullary nail fixation of pertrochanteric fractures: a retrospective study of 813 patients. Arch Orthop Trauma Surg. 2018;138(3):351-359.
24. Kim KH, Han KY, Kim KW, Lee JH, Chung MK. Local Postoperative Complications after Surgery for Intertrochanteric Fractures Using Cephalomedullary Nails. Hip Pelvis. 2018;30(3):168-174.
25. Swaroop S, Gupta P, Bawari R, Marya SK, Patnaik S. Factors Affecting the Outcome of Unstable Intertrochanteric Fractures Managed With Proximal Femoral Nail Anti-Rotation 2: A Prospective Outcome Study in Elderly Indian Population. Cureus. 2020;12(12):e11973.
26. Hancioglu S, Gem K, Tosyali HK, Okcu G. Clinical and Radiological Outcomes of Trochanteric AO/OTA 31A2 Fractures: Comparison between Helical Blade and Lag Screw - A Retrospective Cohort Study. Z Orthop Unfall. 2020.

27. Jamshad OP, Mathew J, Karuppal R. Functional and Radiological Outcome of Unstable Intertrochanteric Fracture in Old Age Treated with Proximal Femoral Nail Antirotation-2. Journal of Clinical and Diagnostic Research. 2021;15(4):RC05-RC07.
28. Barlow E, Larsen MP, Vijayasurej K, Burczy M, Burczy J, Mullins M. The impact of COVID-19 on dynamic hip screw fixation and training. Pol Merkur Lekarski. 2021;49(292):273-277.
29. Cordero-Ampuero J, Peix C, Marcos S, Cordero GGE. Influence of surgical quality (according to postoperative radiography) on mortality, complications and recovery of walking ability in 1425 hip fracture patients. Injury. 2021;52 Suppl 4:S32-S36.
30. Cepni S, Subasi IO, Sahin A, Bozkurt I, Firat A, Kilicarslan K. Tip-neck distance ratio as a novel predictor for failure in cephalomedullary nailing of unstable trochanteric fractures (UTF). Arch Orthop Trauma Surg. 2021.
31. Yamamoto N, Tomita Y, Noda T, Inoue T, Mochizuki Y, Ozaki T. Reduction quality and nail fixation ratio as bone-implant stability factors associated with reoperation for trochanteric fractures. Injury. 2021;52(7):1813-1818.
32. Momii K, Fujiwara T, Mae T, et al. Risk factors for excessive postoperative sliding of femoral trochanteric fracture in elderly patients: A retrospective multicenter study. Injury. 2021;52(11):3369-3376.
33. Hwang J, Hadeed M, Sapp T, et al. Varus displacement of intertrochanteric femur fractures on injury radiographs is associated with screw cutout. Eur J Orthop Surg Traumatol. 2021;31(4):683-687.
34. Shon OJ, Choi CH, Park CH. Factors Associated with Mechanical Complications in Intertrochanteric Fracture Treated with Proximal Femoral Nail Antirotation. Hip Pelvis. 2021;33(3):154-161.
35. Yamamoto N, Imaizumi T, Noda T, Inoue T, Kawasaki K, Ozaki T. Postoperative computed tomography assessment of anteromedial cortex reduction is a predictor for reoperation after intramedullary nail fixation for pertrochanteric fractures. Eur J Trauma Emerg Surg. 2022;48(2):1437-1444.
36. Goto K, Murakami T, Saku I. Postoperative subtype P as a risk factor for excessive postoperative sliding of cephalomedullary nail in femoral trochanteric fractures in old patients: A case series of 263 patients using computed tomography analysis. Injury. 2022.

Appendix B Table S3. Risk of bias judgment in the included studies.

Study	Risk of bias						
	Study participation	Study attrition	Prognostic factor measurement	Outcome measurement	Study confounding	Statistical analysis and reporting	Overall
Baumgaertner 1997	High	Low	Low	Low	High	Moderate	High
Kawaguchi 1998	High	Low	Low	Low	High	Moderate	High
Pervez 2004	High	Low	Low	Low	High	Moderate	High
Hsueh 2010	Moderate	Low	Low	Low	High	Moderate	Moderate
Ertürer 2012	High	Low	Moderate	Moderate	High	Moderate	High
De Bruijn 2012	Low	Low	Low	Low	Moderate	Low	Moderate

Gunay 2014	High	Low	Low	Moderate	High	Moderate	High
Takigawa 2014	Moderate	Low	Low	Moderate	High	Moderate	Moderate
Mingo-Robinet 2015	Low	Low	Low	Moderate	High	Low	Moderate
Fang 2015	Low	Low	Low	Moderate	Low	Low	Moderate
Chang 2015	Low	Low	Low	Low	High	Moderate	Moderate
Ito 2015	Low	Low	Low	Low	High	Moderate	Moderate
Temiz 2015	Moderate	Low	Low	Low	High	Moderate	Moderate
Hsu 2016	Low	Low	Low	Low	Low	Low	Low
Li 2016	Low	Low	Low	Low	Low	Low	Low
Buyukdogan 2016	Low	Low	Low	Low	High	Low	Moderate
Karampinas 2016	Low	Low	Low	Moderate	High	Moderate	Moderate
Caruso 2017	Low	Low	Low	Low	High	Low	Moderate
Ciufo 2017	Low	Low	Low	Low	Moderate	Low	Moderate
Fujii 2017	Moderate	Low	Low	Low	Moderate	Low	Moderate
Aicale 2018	High	Low	Low	Moderate	High	Moderate	High
Morvan 2018	Low	Low	Low	Low	High	Low	Moderate
Murena 2018	Low	Low	Low	Low	High	Low	Moderate
Kim 2018	Low	Low	Moderate	Low	Low	Low	Moderate
Swaroop 2020	High	Low	Low	Moderate	High	Moderate	High
Hancioglu 2020	Moderate	Low	Low	Moderate	High	Moderate	Moderate
Lakho 2021	Low	Low	Low	Moderate	High	High	High
Barlow 2021	High	Low	Low	Low	High	Moderate	High
Cordero-Ampuero 2021	Moderate	Moderate	Moderate	Moderate	High	Moderate	High
Momii 2021	Low	Low	Low	Low	Low	Low	Low
Çepni 2021	Low	Low	Low	Low	High	Low	Moderate

Hwang 2021	Low	Low	Low	Low	High	Low	Moderate
Yamamoto 2021	Low	Low	Low	Low	High	Low	Moderate
Shon 2021	Moderate	Low	Low	Low	High	Low	Moderate
Yamamoto 2021	Low	Low	Low	Low	High	Moderate	Moderate
Goto 2022	Low	Low	Low	Low	High	Low	Moderate

Appendix C Figure S1. Forest plots showing the association between poor reduction by Baumgaertner criteria and failed internal fixation.

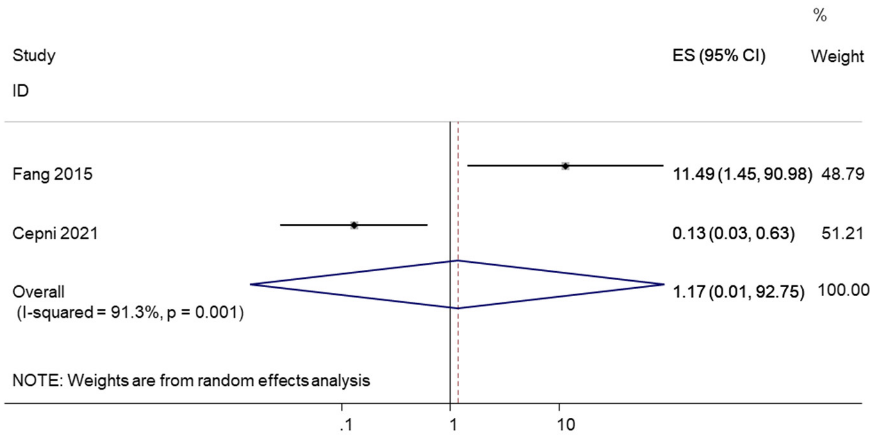

Appendix C Figure S2. Forest plots showing the association between poor reduction by Baumgaertner criteria and cut-out.

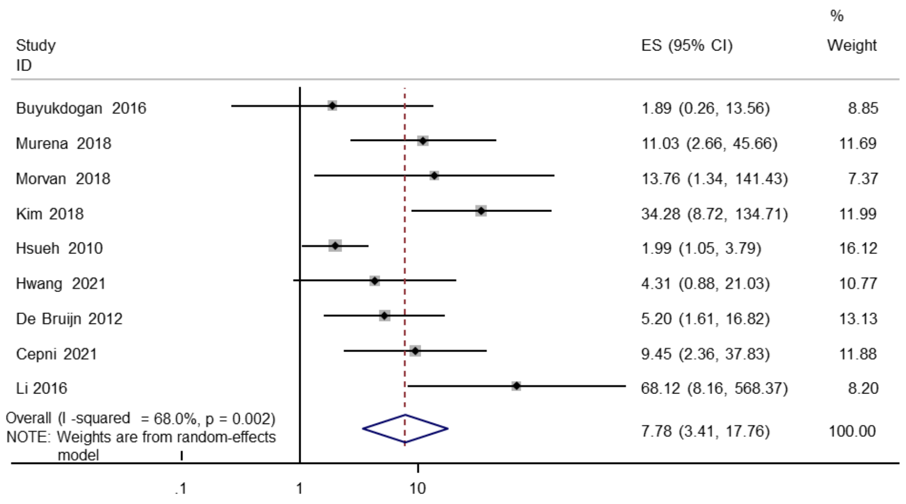

Appendix C Figure S3. Forest plots showing the association between varus malreduction and failed internal fixation.

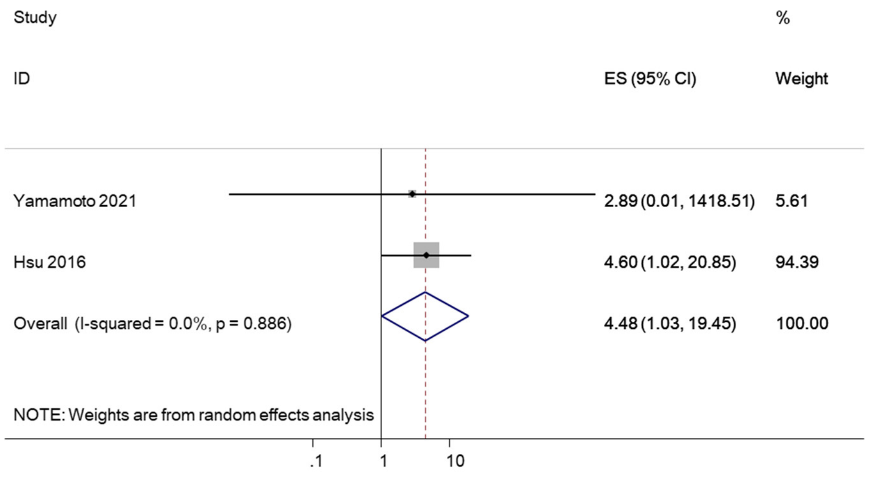

Appendix C Figure S4. Forest plots showing the association between varus malreduction and cut-out.

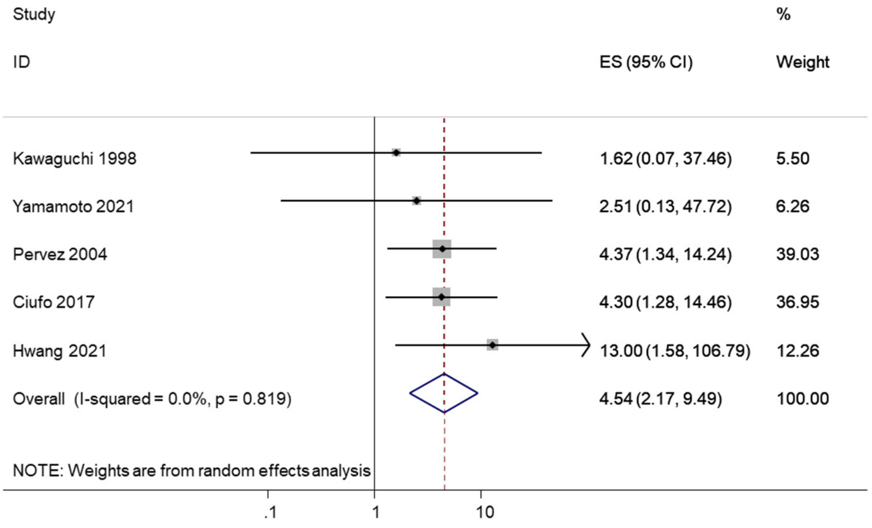

Appendix C Figure S5. Forest plots showing the association between intramedullary malreduction on anteromedial cortex and failed internal fixation.

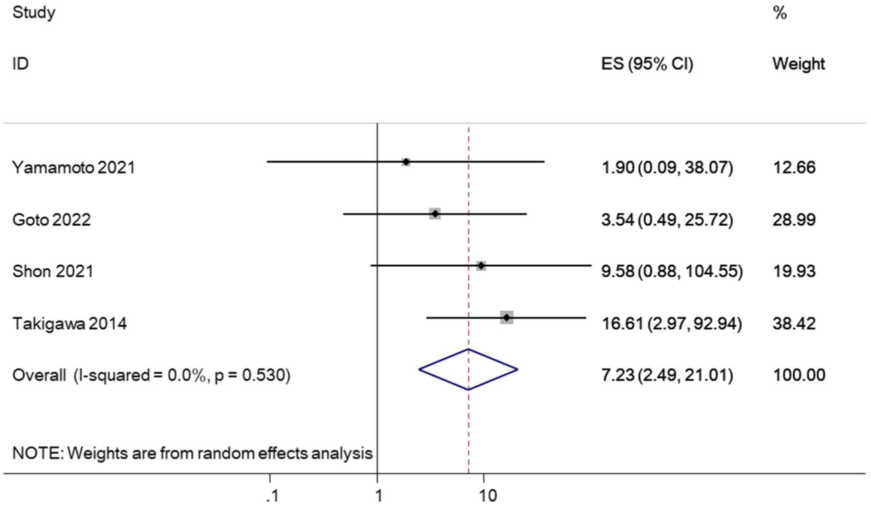

Appendix C Figure S6. Forest plots showing the association between intramedullary malreduction on anteromedial cortex and cut-out.

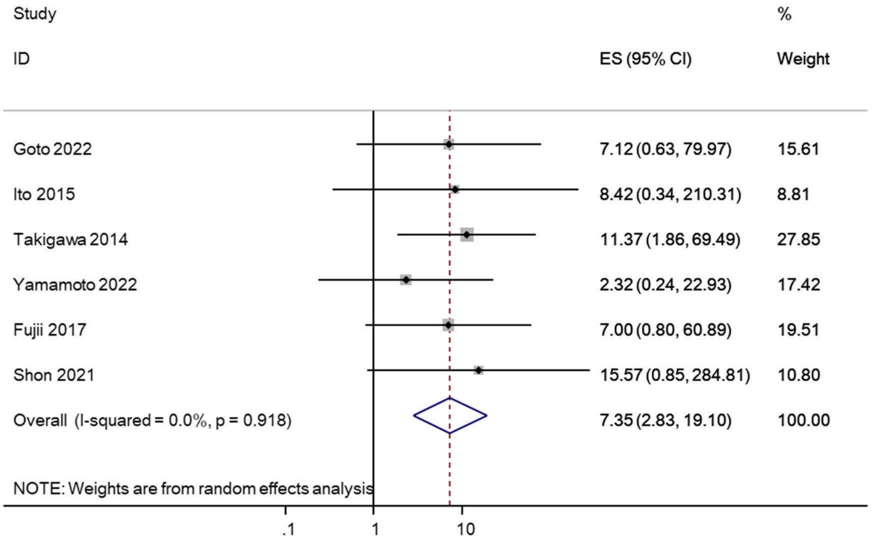

Appendix C Figure S7. Forest plots showing the association between TAD ≥ 25 and failed internal fixation.

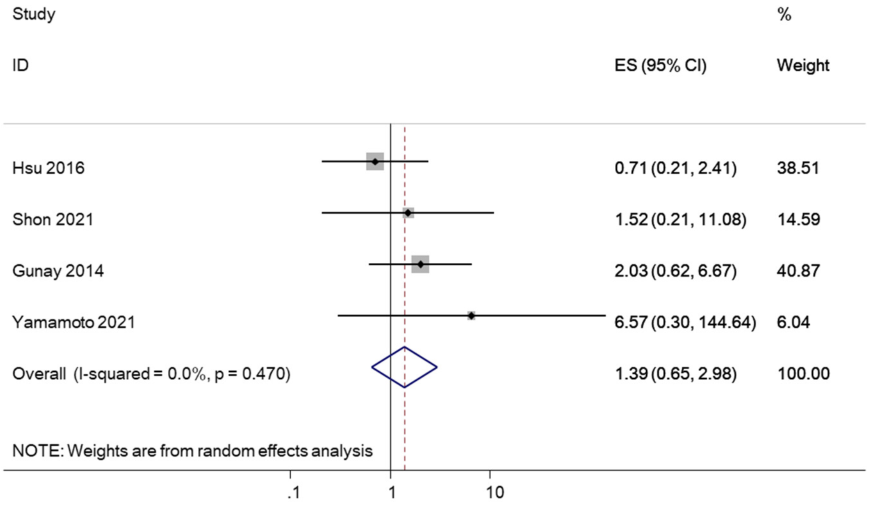

Appendix C Figure S8. Forest plots showing the association between TAD ≥ 25 and cut-out.

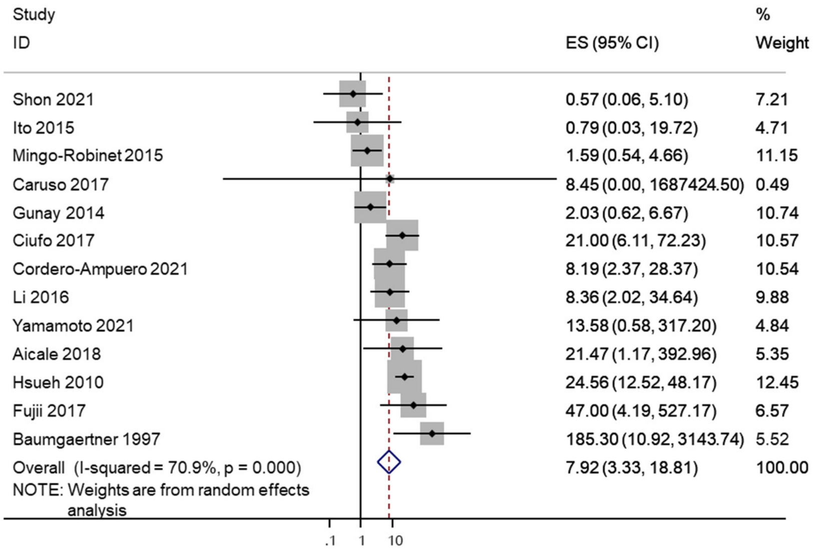

Appendix C Figure S9. Forest plots showing the association between inadequate screw placement in femoral head and failed internal fixation.

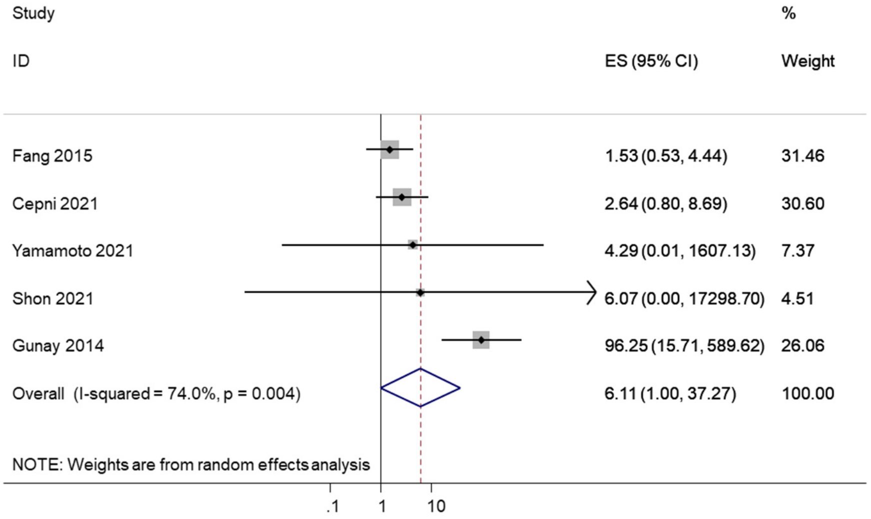

Appendix C Figure S10. Forest plots showing the association between inadequate screw placement in femoral head and cut-out.

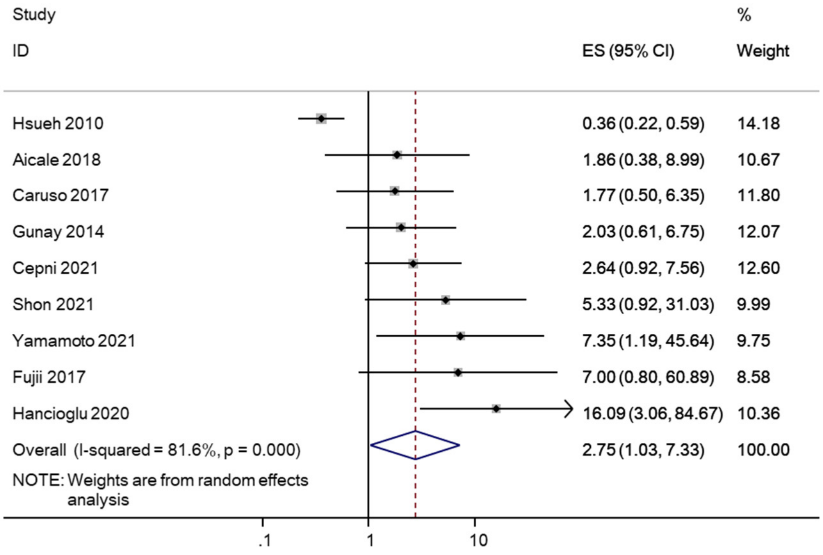

Appendix C Figure S11. Funnel plot of estimates from the included studies on the association between poor reduction by Baumgaertner criteria and cut-out.

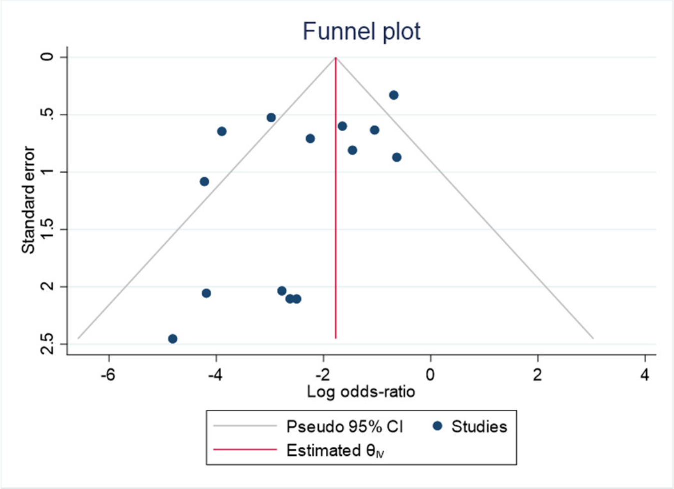

Appendix C Figure S12. Funnel plot of estimates from the included studies on the association between TAD ≥ 25 and cut-out.

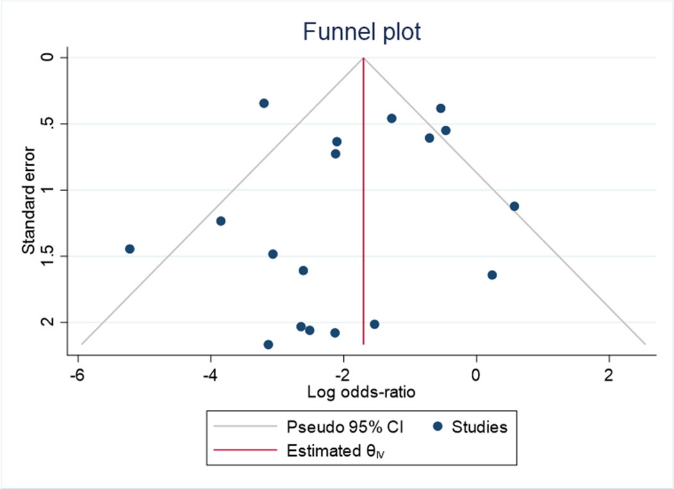

Appendix C Figure S13. Funnel plot of estimates from the included studies on the association between inadequate screw placement in femoral head and cut-out.

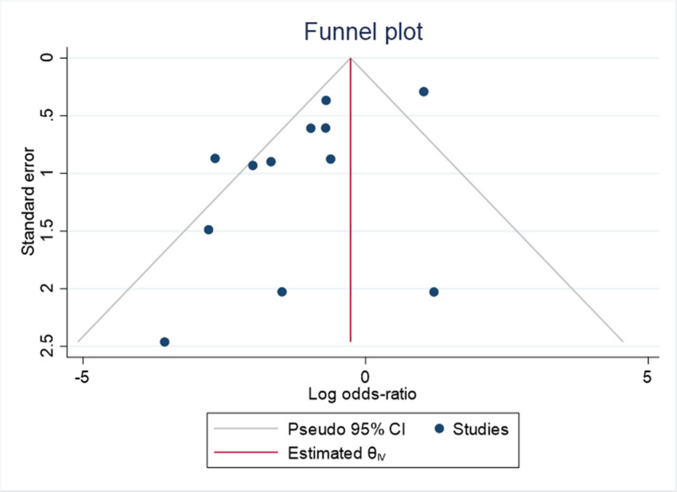

Appendix C Figure S14. Subgroup analysis of implant type in the association between poor reduction by Baumgaertner criteria and cut-out.

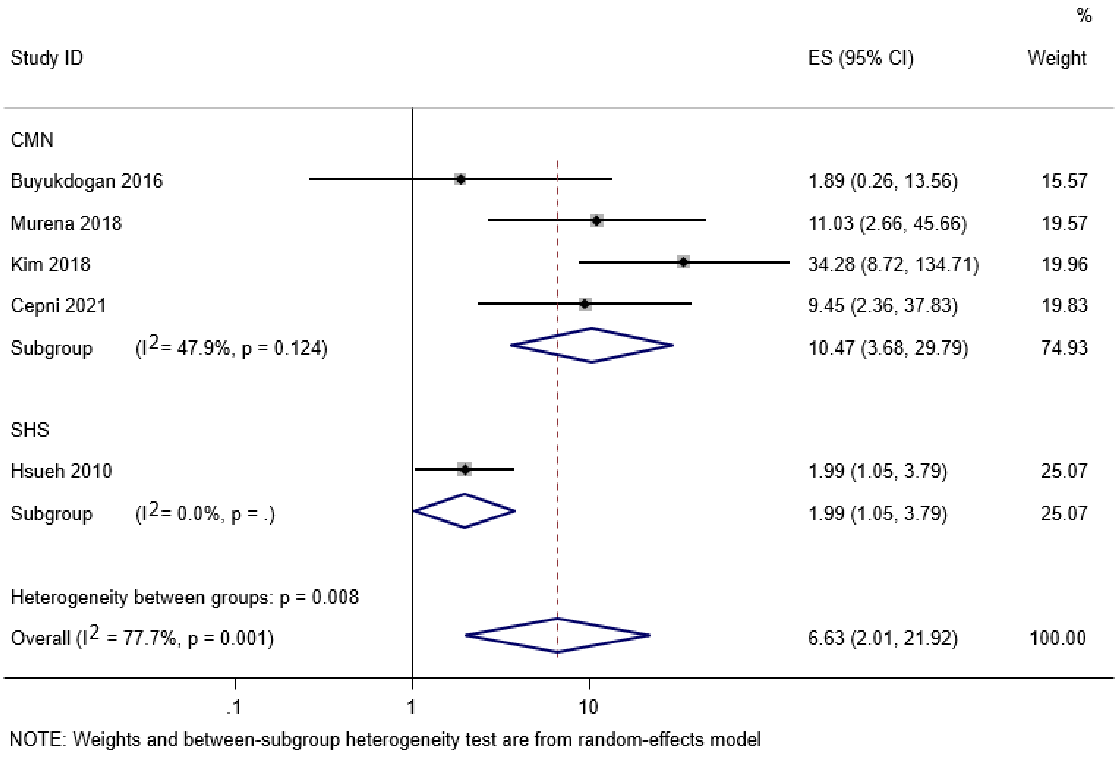

Appendix C Figure S15. Subgroup analysis of implant type in the association between varus malreduction and cut-out.

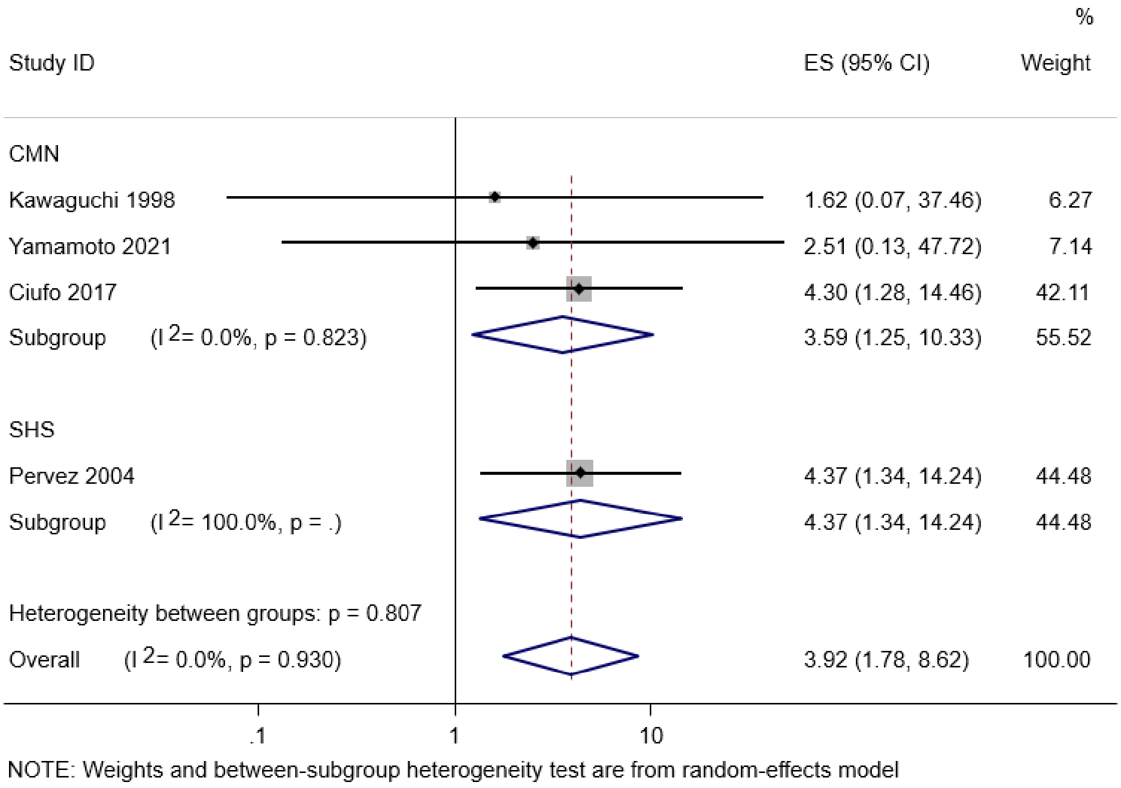

Appendix C Figure S16. Subgroup analysis of implant type in the association between TAD ≥ 25 and failed internal fixation.

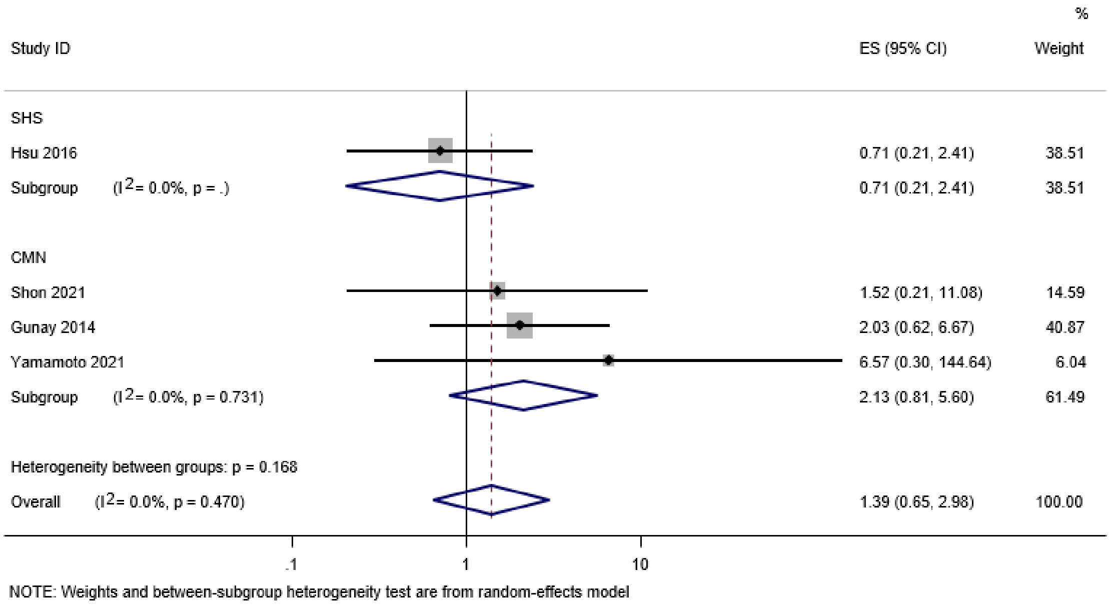

Appendix C Figure S17. Subgroup analysis of implant type in the association between TAD ≥ 25 and cut-out.

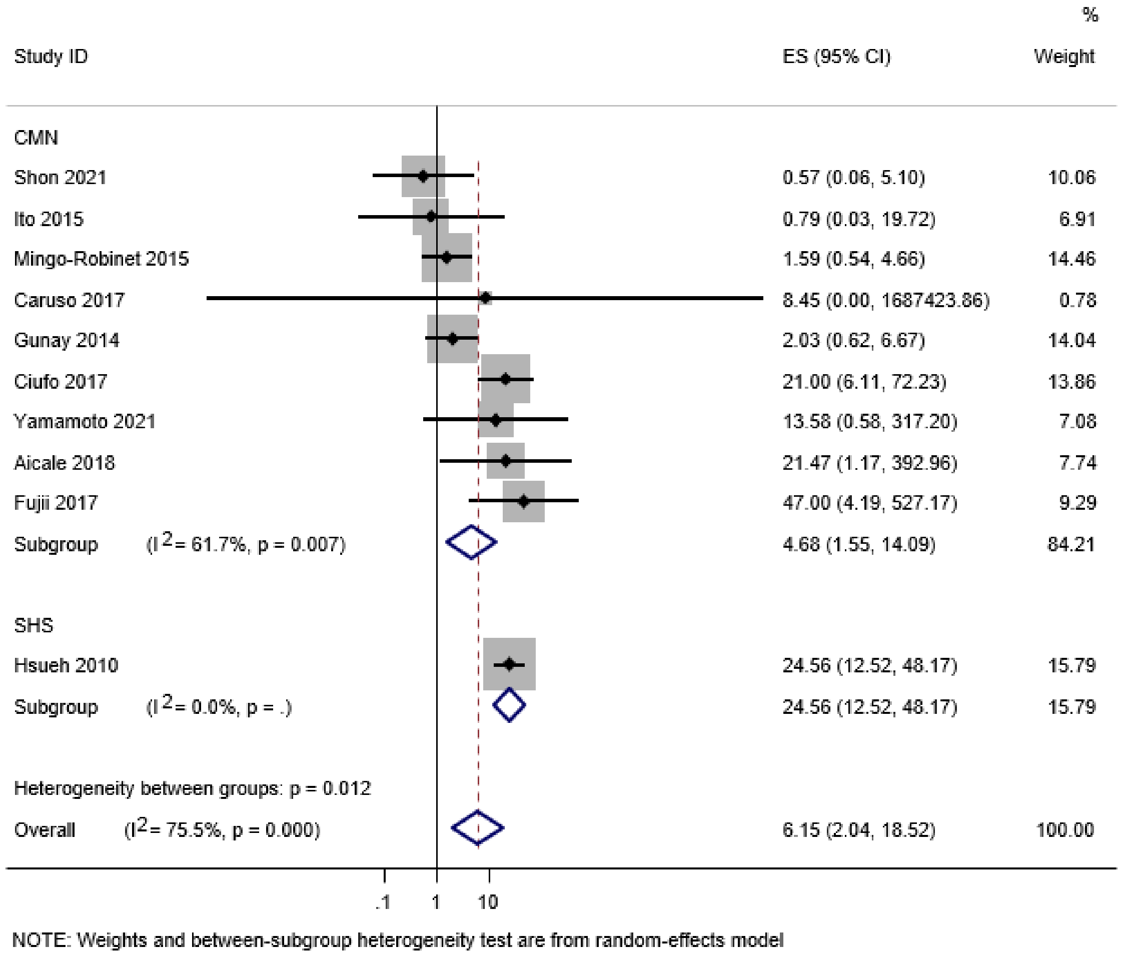

Appendix C Figure S18. Subgroup analysis of implant type in the association between inadequate screw placement in femoral head and failed internal fixation.

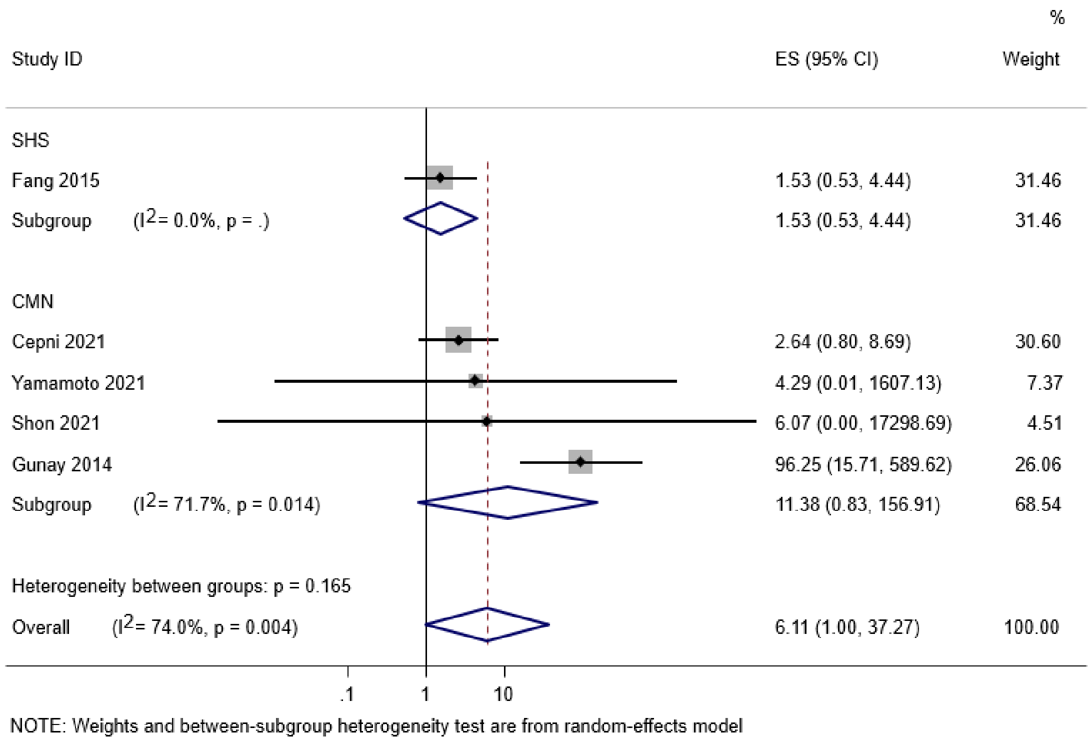

Appendix C Figure S19. Subgroup analysis of implant type in the association between inadequate screw placement in femoral head and cut-out.

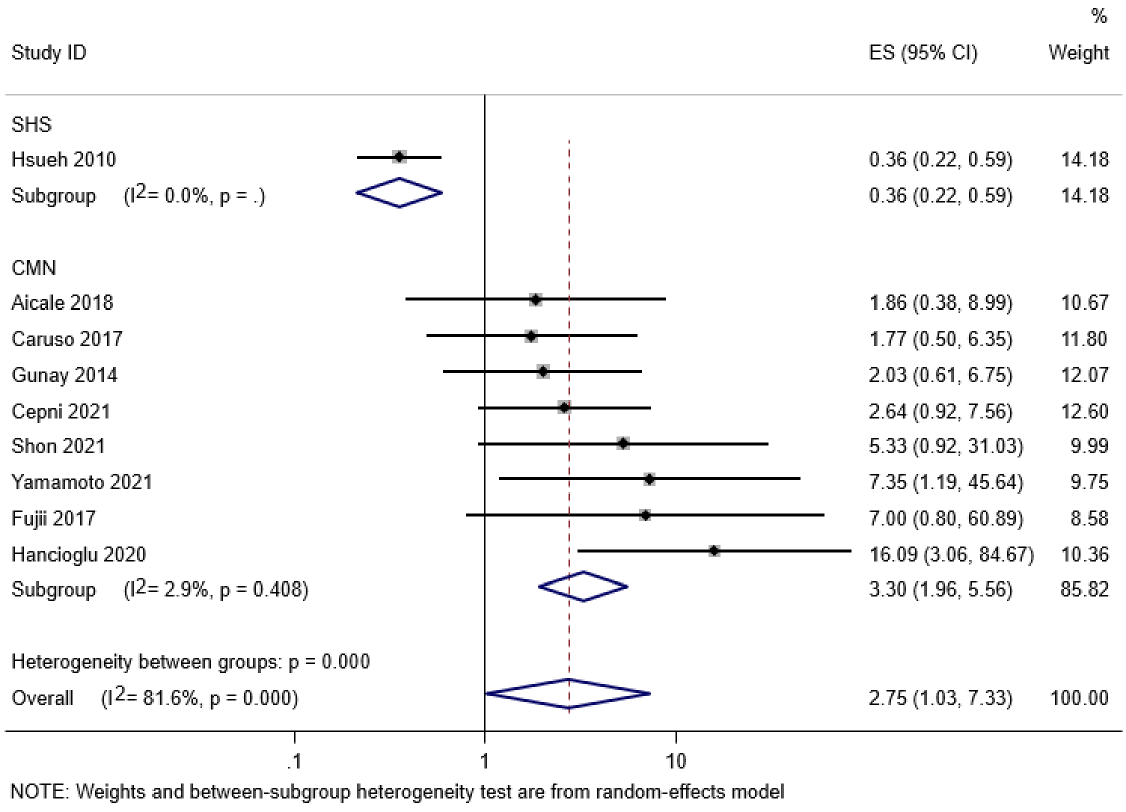

Appendix C Table S4. Subgroup analysis on association of immediate postoperative radiographic findings and failed internal fixations; fracture type.

		Unstable fracture types		
Prognostic factors	Outcomes	No of studies	I^2 (%)	Pooled odds ratio (95% CI)
Poor reduction by Baumgaertner criteria	Failed internal fixation	1	-	9.46 (2.36 to 37.8)
	Cut-out	1	-	9.46 (2.36 to 37.8)
Varus malreduction	Failed internal fixation	-	-	-
	Cut-out	-	-	-
Intramedullary malreduction	Failed internal fixation	-	-	-
	Cut-out	-	-	-
TAD ≥ 25	Failed internal fixation	-	-	-
	Cut-out	1	-	8.19 (2.37 to 28.37)
Inadequate screw placement in femoral head	Failed internal fixation	1	-	2.64 (0.80 to 8.69)
	Cut-out	1	-	5.78 (1.00 to 33.50)

CI, confidence interval; TAD. tip-apex distance
